# ﻿Morphological and molecular revision of the subfamily Heterolepismatinae (Zygentoma, Lepismatidae), with descriptions of two new genera from the Atacama Desert, Chile

**DOI:** 10.3897/zookeys.1260.151902

**Published:** 2025-11-20

**Authors:** Rafael Molero-Baltanás, Álvaro Zúñiga-Reinoso, Miquel Gaju-Ricart, Reinhard Predel

**Affiliations:** 1 Department of Zoology, University of Córdoba, C-1 Campus de Rabanales, 14071 Córdoba, Spain University of Córdoba Córdoba Spain; 2 Institute of Zoology, University of Cologne, Zülpicher Str. 47b, 50674 Köln, Germany University of Cologne Köln Germany

**Keywords:** *
Cactisma
*, *
Heterolepisma
*, *
Lapidisma
*, silverfish, South America, *
Visma
*, *
Vistrolepisma
*

## Abstract

This work aims to update the systematics of silverfish of the subfamily Heterolepismatinae (Zygentoma, Lepismatidae) based on morphological and molecular data of new taxa from the Atacama Desert (Chile) with a comparison with specimens from Argentina, from where species originally described as belonging to the genus *Heterolepisma* Escherich, 1905 come. This comparison also includes COI gene sequences of Argentine specimens attributed to *H. andinum* (Silvestri, 1902), and of Australian Heterolepismatinae previously classified as *Heterolepisma* or *Visma* Smith et al., 2021. As a result, the genera *Lapidisma***gen. nov.** and *Cactisma***gen. nov.** are described from Chile, and new morphological characters diagnostic of *H. andinum* are provided. The species *Lapidisma paposanum***sp. nov.** and *Cactisma camanchaca***sp. nov.** are associated with different habitats. The species *Heterolepisma annectens* (Silvestri, 1924) is tentatively attributed to the first genus (*Lapidisma annectens***comb. nov.**). The molecular data place the three South American genera in separate lineages, but cluster them as a sister group to the Australian *Heterolepisma* + *Visma*. This supports the proposal of the genus *Silvestrisma***gen. nov.** to include Australian species previously assigned to *Heterolepisma*. Additionally, the new name *Vistrolepisma***nom. nov.** is proposed for silverfish species previously assigned to the genus *Visma*, since this generic name is pre-occupied. With this re-arrangement, the subfamily Heterolepismatinae consists of six genera, pending further studies in other geographic regions where poorly studied species occur that were (and are provisionally) assigned to *Heterolepisma*.

## ﻿Introduction

Heterolepismatinae Mendes, 1991 is a subfamily of silverfish (Order Zygentoma, family Lepismatidae) widespread in the Southern Hemisphere, probably with a Gondwanan origin (Molero-Baltanás et al. 2024). It was defined by Mendes (1991) and the most remarkable characters that he provided for the diagnosis (not necessarily synapomorphic characters) of this subfamily are the smooth macrochaetae, apically bifid or trifid; apical article of maxillary palp with few cylindrical sensilla (the pronged apical sensilla of Molero-Baltanás et al. 2024), in some species with sexual dimorphism; apical article of labial palp with 3+2 papillae arranged in two rows; anterior margin of pronotum with setal collar; thoracic sternites well developed; parameres vesiculiform, lacking glandular area. More recently, Mendes (2018) considered a greater variability of the parameres for this subfamily (also subcylindrical and pseudo-articulated).

All species belonging to Heterolepismatinae had been included in the genus *Heterolepisma* Escherich, 1905, until Smith and Mitchell (2019) and Smith et al. (2021) distinguished the genera *Maritisma* Smith & Mitchell, 2019 and *Visma* Smith, Mitchell & Molero-Baltanás, 2021, respectively; both with representatives only in Australia and neighbouring Pacific islands, with the exception of *Maritisma dispar* (Yosii, 1939), described from Japan as *Heterolepisma dispar*. The generic name *Visma* is pre-occupied for a gastropod belonging to the family Pyramidellidae and the silverfish formerly known by this name receives a new name here. Therefore, *Visma* referring to Lepismatidae is only used before the Results section. Excluding the species of the above-mentioned two new genera, the genus *Heterolepisma* currently consists of 27 species (see first column in Table 1).

**Table 1.** List of silverfish species previously ascribed to the genus *Heterolepisma*. Species that were already transferred to the genera *Maritisma* and *Vistrolepisma* (formerly *Visma*) prior to our study are not considered here. *Heterolepisma trisetosum* is not included as it is considered a species inquirenda (see Discussion); species highlighted in bold require revision to confirm generic assignment; * indicates insufficient original description without updated redescription.

Species, author(s), and year of description	Distribution	Remarks (original names, synonyms, proposed generic status, etc.)
*H. andinum* (Silvestri, 1902)*	Argentina	Described as *Lepisma andina*
***H. annectens*** (Silvestri, 1924)*	J. Fernández Islands	Provisionally transferred in this work to *Lapidisma annectens* comb. nov.
***H. bisetosum*** (Carpenter, 1916)*	Seychelles, Somalia	Redescription by Mendes (1988) did not provide enough information for updating generic status
*H. buntonorum* Smith, 2016	Tasmania (Australia)	*Silvestrisma buntonorum* comb. nov.
*H. cooloola* Smith, Mitchell, Lee & Espinasa, 2019	Australia (QLD)	*Silvestrisma cooloola* comb. nov.
*H. coorongooba* Smith, Mitchell, Lee & Espinasa, 2019	Australia (NSW)	*Silvestrisma coorongooba* comb. nov.
***H. exactum*** (Silvestri, 1918)*	Zanzíbar (Tanzania)	Described as *Isolepisma exacta*
*H. heraldense* Smith & Mitchell, 2019	Australia (Coral Sea T.)	*Silvestrisma heraldense* comb. nov.
*H. highlandi* Smith, 2014	Australia (NSW)	*Silvestrisma highlandi* comb. nov.
***H. horni*** Stach, 1933*	Ecuador (Caracas Bay) Caribbean	Stach (1933) provided an identification key of the species known at that time
*H. howense* Womersley, 1942	Lord Howe Island (Australia)	*Silvestrisma howense* comb. nov., Redescription by Smith and Mitchell (2019)
***H. insulare*** (Banks, 1901)*	Galapagos Isl. (Ecuador)	Described as *Lepisma insularis* = *H. intermedium* Folsom, 1924
***H. japonicum*** (Uchida, 1968)	Japan	Described as *Isolepisma japonica*
*H. kraepelini* Silvestri, 1908*	Australia (SA, WA)	(*Silvestrisma kraepelini* comb. nov.)
*H. michaelseni* Silvestri, 1908*	Australia (SA, WA)	(*Silvestrisma michaelseni* comb. nov.)
*H. milledgei* Smith & Mitchell, 2019	Lord Howe Island (Australia)	(*Silvestrisma milledgei* comb. nov.)
***H. mossambicense*** Mendes, 1993	Mozambique	Described as H. *mossambicensis*
***H. mumfordi*** (Silvestri, 1935)*	Marquesas Isl. (Fr. Polynesia)	Described as *Isolepisma mumfordi*
*H. pampeanum* (Silvestri, 1902)*	Argentina	Type species of the genus. Wygodzinsky (1948) gave some characters, but without completing the description
*H. parvum* Smith, 2013	Barrow Island (Australia)	*Silvestrisma parvum* comb. nov., described as *Heterolepisma parva*
***H. primafrum*** (Silvestri, 1949)	Angola	Described as *Isolepisma primafra*
*H. pyramidum* Smith & Mitchell, 2019	Lord Howe Island (Aust.)	*Silvestrisma pyramidum* comb. nov.
***H. rouxi*** (Silvestri, 1915)*	New Caledonia	Described as *Isolepisma rouxi*
*H. sclerophyllum* Smith, 2014	Australia (NSW)	*Silvestrisma sclerophyllum* comb. nov., described as *Heterolepisma sclerophylla*
***H. serranoi*** Mendes, 2011	Brazil (Paraiba)	
***H. tonga*** Mendes, 2012	Tonga	
***H. zelandicum*** (Tillyard, 1924)	New Zealand	After the very incomplete original description as *Notolepisma zelandica*, Wygodzinsky (1961) provided a re-description but it does not provide sufficient details for an updated generic description

The type species of *Heterolepisma* is *H. pampeanum* (Silvestri, 1902), described from several localities in Argentina as *Lepisma pampeana* Silvestri, 1902 and transferred to the genus *Heterolepisma* when it was erected (Escherich 1905). Another Argentine species belonging to this genus is *H. andinum* (Silvestri, 1902). From continental Chile there are no records of *Heterolepisma* species, but one species, *H. annectens* (Silvestri, 1924), was described from the archipelago of Juan Fernández (Silvestri 1924). Some authors transferred all *Heterolepisma* to *Isolepisma* Escherich, 1905, a genus created for *I. trisetosum* Escherich, 1905, but Silvestri considered both genera as synonyms and, consequently, they have been treated by most authors during the last 60 years as *Heterolepisma*. According to Mendes (2011), *I. trisetosum* should be treated as species inquirenda.

A comprehensive survey of insects across the Atacama Desert as part of the project “Earth and Evolution at the dry limit” (https://sfb1211.uni-koeln.de/) revealed several undescribed taxa of Zygentoma (for *Maindronia* Bouvier, 1897, see Zúñiga-Reinoso and Predel 2019). Based on molecular and morphological data, we here describe two new genera of the poorly studied subfamily Heterolepismatinae and discuss the relationships of the genera currently included in this subfamily.

## ﻿Materials and methods

Sampling of the type series and genetically similar specimens from neighbouring areas took place between August – November 2023 along the coastal section of the Atacama Desert in Antofagasta Province (Chile) around Paposo and Cuesta el Cobre (sampling permits CONAF N° 016/2021, 000319°N). Additionally, specimens assigned to *Heterolepisma* were collected near Sanogasta, La Rioja (Argentina; 29°21.03'S, 67°46.93'W, sampling permit of the Secretaría de Ambiente: 244/22) and Talcahuano in Southern Chile (36°42.52'S, 73°7.05'W). Specimens were collected using an aspirator and directly transferred into 96% ethanol.

### ﻿DNA extraction, amplification, and sequencing

From the type specimens, DNA was extracted from legs or remaining tissues without gut after the dissection and preparation of the exoskeleton for morphological analyses (marked with “*” in the examined material section). Additionally, DNA was extracted from the head and pronotum of further specimens. DNA extraction was performed using a modified protocol of the EZNA Insect DNA Kit (Omega Bio-tek, Inc., Norcross, U.S.A). A fragment of the mitochondrial gene cytochrome oxidase I (COI) was amplified using the primers LCO1490COL* (WYTCDACWAAYCRYAARGAYATYGG) and C1-N-2329COL* (ACTGTAAATATRTGATGWGCTCA). The thermal polymerase chain reaction (PCR) steps were 94 °C for 2 min, followed by 36 cycles at 94 °C for 30 s, 53 °C for 45 s, and 72 °C for 1 min, with a final extension at 72 °C for 2 min. The PCR products were purified using peqGOLD Cycle-Pure kit (Peqlab Biotechnologie GmbH, Erlangen, Germany), and both forward and reverse products were sequenced at Eurofins Genomics GmbH (Germany) using the Sanger sequencing method. The sequences were edited in BIOEDIT 7.0.5.3 (Hall 1999) and checked with BLAST to confirm that the sequences belong to Zygentoma. COI sequences of other Zygentoma (Heterolepismatinae: *Visma*, *Heterolepisma*; Ctenolepismatinae: *Thermobia* Bergroth, 1890, *Ctenolepisma* Escherich, 1905, *Hemitelsella* Smith, 2016, *Qantelsella* Smith, 2015; Lepismatinae; *Neoasterolepisma* Mendes, 1988, *Lepisma* Linnaeus, 1758; Maindroniidae: *Maindronia*) available in GenBank were selected to construct a more comprehensive matrix for a phylogenetic analysis. DNA sequences were reviewed, orthologous sequences were aligned using the Clustal W (Thompson et al. 1994) algorithm implemented in BIOEDIT 7.0.5.3, and then manually checked for inconsistencies. The alignment is available in Suppl. material 1. Subsequently, we calculated the genetic distance under the model p-distance for all taxa in MEGA 7 v. 26 (Kumar et al. 2016).

### ﻿Phylogenetic reconstruction

The aligned matrix was used for the phylogenetic reconstructions using Bayesian inference (BI) algorithms. The sequence for *Maindronia* (undescribed species belonging to clade A in Zúñiga-Reinoso and Predel 2019; GenBank accession number: MN218531) was used to root the phylogenetic tree. Selection of substitution models for the phylogenetic analysis was done using Bayesian information criteria with the software Modelfinder v. 2.0 (Kalyaanamoorthy et al. 2017) implemented on IQTREE web server (Trifinopoulos et al. 2016). Selected information was used as a prior setting in a Mr. Bayes block. The BI was performed with the program Mr. Bayes 3.2.6 (Ronquist et al. 2012) implemented in the server CIPRES Science Gateway 3.3 (Miller et al. 2010). Four separate runs were conducted using four chains. Each run initiated with a randomly generated tree, for 10 million generations, and sampled every 1000 trees. The initial 25% of the trees generated were excluded as burn-in. Once the convergence of these four independent runs was verified based on metrics like the average standard deviation of split frequencies and the potential scale reduction factor, the outcomes from these runs were combined, resulting in a total of 90,004 trees. Lastly, a consensus tree was created by applying a 50% majority rule, with node assessments determined using posterior probability (pp).

### ﻿Morphological study

Insects preserved in ethanol (96%) were dissected, mounted on slides using Tendeiro Medium and examined using an Axioskop 2 plus microscope (Carl Zeiss GmbH, Göttingen, Germany), equipped with a Zeiss AxioCam MRm camera. Figures of morphological characters were drawn based on these photographs, with the help of GIMP2 software. For the description of morphological characters, we used the terminology of Smith (2013) and Molero-Baltanás et al. (2024); for example, the term ‘comb’ (of macrochaetae) is used in the sense indicated in these works, i.e., “rows of macrochaetae that are more or less perpendicular to the lateral margin. They can be composed of few (2 or 3) or a higher number of macrochaetae each” and “in some cases, a comb may be reduced to a single macrochaeta but its submarginal position makes it clear that this represents a reduced number of macrochaeta in a normally positioned comb and it is counted as a comb.” For the description of the position of trichobothria on thoracic nota and for the types of antennal sensilla, the suggestions of Smith et al. (2021) and the terminology of Adel (1984) were adopted, respectively. For cylindrical sensilla of the apical article of maxillary palps (Mendes 1991), in silverfish taxonomy also referred to as pronged sensilla (see Molero-Baltanás et al. 2024), we use the term styloconic sensilla; they consist of several apical sensory cones (short and apically subacute or longer and finger-shaped) that insert into a cylindrical base (style). Details of some specimens were examined with a Zeiss Sigma 300-VP scanning electronic microscope (SEM). Previous to the SEM examination, insects stored in 96% ethanol were dehydrated as follow: 2h absolute ethanol followed by 3 × 2h hexamethyldisilazane (HMDS), finally evaporation of HMDS at room temperature. Dried samples were directly placed on carbon adhesive discs (Plano, Wetzlar, Germany) on the specimen holder and subsequently sputter-coated with gold layers. Prior to dehydration, some specimens were ultrasonically cleaned, which generally resulted in a loss of most scales and macrochaetae. The type material is deposited in the following institutions:

**MNNC** Museo Nacional de Historia Natural, Santiago (Chile);

**MZUC** Museo de Zoología de la Universidad de Concepción, Concepción (Chile);

**UCO** Departamento de Zoología, Universidad de Córdoba, Córdoba (Spain).

## ﻿Results

### ﻿Taxonomic account

#### ﻿ Lapidisma gen. nov.

Taxon classificationAnimaliaZygentomaLepismatidae

9D4D17FF-CCA5-596A-9726-21BD9E914EFE

https://zoobank.org/E9D457F2-A04C-4FA5-A107-855277955B0C

##### Type species.

*Lapidisma paposanum* sp. nov.

##### Diagnosis.

Medium-sized silverfish (adults reaching 7.0–8.5 mm). Body shape fusiform, with thorax slightly wider than the abdomen base. Antennae as long as body or a little longer; caudal filaments slightly shorter than body. Epidermal pigment present, dorsal scales pigmented. Multi-radiate orbicular scales, rounded to subquadrangular, covering the body dorsally and ventrally, also present on coxae and clypeus. Scales of femora and tibiae ovoid, wide, smaller than those of coxae and body, with their base not orbicular (not surrounding the socket). Subtriangular apically truncate scales present on scape and pedicel of antennae and basal articles of maxillary palps; some scales showing this shape are also present on femora. Scales absent from labrum, apical articles of maxillary palps, labial palps, tarsi, and abdominal styli; caudal filaments with some narrow lanceolate scales. Macrochaetae smooth, apically bifurcate. Compound eyes with 12 ommatidia. Frontal chaetotaxy concentrated on lateral parts, with a wide gap in the middle. Clypeus almost vertical, with 1+1 lateral tufts of macrochaetae and rounded scales. Labrum folded backwards, with a transverse group of macrochaetae not forming clear lateral tufts. Antennae with trichobothria and also with chaetic, trichoid, coeloconic and several types of basiconic sensilla. Galea with a small apical peg. Maxillary palp with the apical article bearing basiconic sensilla types B and C, and a longitudinal row of three styloconic sensilla. Apical article of the labial palp with five typical papillae arranged in a jagged line, not clearly forming two lines, nor a straight one; the outer lateral area with basiconic sensilla types B and C. Anterior margin of pronotum with setal collar. Lateral margins of thoracic nota with several macrochaetae, most of them isolated, in some places forming implied combs of two macrochaetae; posterior margin devoid of setae. Trichobothrial areas open, Type 1 according to classification of Mendes (1986). Thoracic sternites parabolic, with convex or slightly truncated hind margin. Coxae with a row of macrochaetae on their anterior margin and on the posterior margin. Metatibiae bearing a long anterior trichobothrium. Tarsi with four articles; pretarsal claws typical, with a short striated medial empodial claw. Urotergites I−VIII with 3+3 combs of macrochaetae; those at submedian position can consist of 1–2+1–2 macrochaetae (when two are present, they are inserted very close together and arranged obliquely to the posterior margin of the tergite). Urotergite IX devoid of setae. Urotergite X short trapezoidal, with hind margin almost straight or with its median part slightly convex, with several setae on their lateral margins, lacking combs. Urosternite I without setae. Urosternites II−VIII in male and II−VII in female with 1+1 single macrochaetae; each coxite VIII of females also with only one isolated macrochaeta. Four pairs of abdominal styli in both sexes. Parameres ovoid or short subcylindrical, with a division delimiting an apical part with some glandular setae. Females with a thin ovipositor of the primary type.

##### Remarks.

The combination of characters indicated in the diagnosis of this genus is more similar to those shared by the Australian species included in the genus *Visma* than to those shared by Australian taxa included in *Heterolepisma*. As the name *Visma* is pre-occupied (see Introduction), the new name assigned here to this genus of Lepismatidae is *Vistrolepisma* nom. nov.; the prefix ‘Vistro’- refers to *vistrum*, a name for a cephalic ‘visor’ consisting of frontal scales, which inspired Smith et al. (2021) to use the name *Visma* for this genus.

Comparison with South American species assigned to *Heterolepisma* is more difficult since specimens in good conditions belonging to these American taxa are not available, with the exception of a recently collected *Heterolepisma* from Argentina that is treated here as *H. andinum*. Similarities and differences with other taxa of Heterolepismatinae are presented in Tables 2, 3. We emphasise that, like *Vistrolepisma*, *Lapidisma* gen. nov. has bifid macrochaetae on labrum, scales on scapus, three slender styloconic sensilla in the apical article of maxillary palp, wide scales on femora and tibiae, and short urotergite X, but it differs from *Vistrolepisma* in lacking 1+1 posterior macrochaetae on the hind margins of thoracic nota, the convex shape of thoracic sternites (truncate in *Vistrolepisma*), a higher number of macrochaetae on infralateral combs, the shape of clypeal scales and the presence of 3+3 combs on urotergite VIII (2+2 in *Vistrolepisma* in this position), although the submedian combs of this urotergite can be reduced to one or two macrochaetae. Looking for affinities with other South American species previously described and comparing the new taxon from Atacama Desert with the species geographically found in the Southern Cone, which is the topotypic area of *Heterolepisma* sensu stricto (s. str.), such as *H. andinum* and *H. pampeanum*, we can clearly distinguish the new taxon from both species because it has a higher number of styli (one pair in *H. andinum* and two pairs in *H. pampeanum*). Moreover, *H. andinum* is different from *Lapidisma* gen. nov. because the latter has only 1+1 lateral macrochaetae on urosternites and *H. andinum* bears 1+1 lateral combs of several macrochaetae, 4–6 according to Stach (1933); *H. pampeanum* is similar in this character to the new species from Atacama Desert. Other characters that have proven to be relevant for generic diagnosis, such as trichobothrial areas, distribution, and shape of scales on appendages, shape and chaetotaxy of thoracic sternites, cephalic chaetotaxy, etc., were not mentioned in the original descriptions of these species or in later published manuscripts that listed some additional characters of the Argentine species (Stach 1933; Wygodzinsky 1948). However, examination of the specimen collected in Argentina, which corresponds to *H. andinum* in the number of styli and abdominal chaetotaxy, suggests that this species probably belongs to a different genus, as it differs from *Lapidisma* gen. nov. in the shape and distribution of scales on appendages, cephalic and ventral chaetotaxy, among other things (see Tables 2, 3). It is likely that most characters of *H. andinum* are shared with *H. pampeanum*, the type species of the genus *Heterolepisma*, although this requires examining the types of this species or, at least, some material identified by experts (such as P. Wygodzinsky) as this species. This could confirm our hypothesis that *Heterolepisma* s. str. is endemic to the eastern side of the Andean mountain range, and that the Andean biogeographic barrier separates *Heterolepisma* s. str. from the Chilean genera described in this work. A re-examination of *H. annectens* from Juan Fernandez Islands may clarify whether this species should be assigned to *Lapidisma* gen. nov., since some shared characters, such as the number of styli, the large size of parameres, and the absence of chaetotaxy on the hind margins of thoracic nota, support this hypothesis. We propose that this species can be considered as *Lapidisma annectens* comb. nov. Compared to *L. annectens*, *L. paposanum* sp. nov. has a higher number of macrochaetae on infralateral combs, but other differences cannot be established because the incomplete description of Silvestri (1902).

**Table 2.** Comparison of cephalic and thoracic morphological characteristics of the new genera of Heterolepismatinae from Chile with *Silvestrisma* gen. nov., *Maritisma*, and *Vistrolepisma* nom. nov. from Australia. For comparison with Heterolepismatinae from other continents (all of them attributed so far to the genus *Heterolepisma* s. str.), see Table 4.

Character	*Lapidisma* gen. nov.	*Cactisma* gen. nov.	*Silvestrisma* gen. nov.	*Vistrolepisma* nom. nov.	* Maritisma *
Median gap on frontal fringe	Yes	Yes	Variable (some species no gap), others with 1+1 macrochaetae in the middle of the gap)	Yes	No
Clypeal scales	Present, rounded (detected with SEM)	Not detected (SEM used)	Not detected or lanceolate	Detected in some species, triangular or lanceolate	Not detected (SEM not used)
Bifid macro-chaetae on labrum	Yes	Yes	No, only small setae	Yes	Yes
Scales on scapus	Wide truncate, subtriangular (SEM)	Not detected (even with SEM)	Absent. Perhaps in some species lanceolate	Triangular or lanceolate (in this case, with an apical indentation)	Not detected (SEM not used)
Scales on pedicel	Triangular, truncate, or even bifid (SEM)	Not detected, even with SEM	Absent (or perhaps lanceolate in some species)	Sometimes, triangular	Absent
Styloconic sensilla on maxillary palp	3, slender styles with several short cones	(2-)3, slender styles with several short cones	Variable, but mostly 3 short styles with several long cones	3, slender styles with several long cones	Not detected, apparently only with a basiconic sensillum C
Scales on maxillary palp	Narrow lanceolate or subtriangular and truncate, on basal article	Not detected, even with SEM	Absent or not detected	Sometimes observed, triangular	Absent or not detected
Papillae on labial palp	5 compact, forming two rows. Apical article wide.	5 compact, forming an oval shape. Apical article narrower.	5 usually compact, forming two more or less defined rows	5 compact, variable arrangement but tending to form only one row	5 not compact, forming two rows in a very wide article
Macrochaetae on posterior margins of thoracic nota	0	0	1–2	1–2	1
Scales on femora and tibiae	Wide, rounded; absent in outer side of tibiae, wider (SEM)	Wide, subtriangular narrower (SEM)	Absent or lanceolate	Triangular to rounded, wide	Absent
Thoracic sternites	Parabolic, with convex to slightly truncate hind margin	Parabolic, with convex hind margin	Triangular to parabolic, usually with convex hind margin	Trapezoidal, with truncate hind margin	Sub-parabolic, with convex hind margin

**Table 3.** Comparison of abdominal morphological characteristics of the new genera of Heterolepismatinae from Chile with *Silvestrisma* gen. nov., *Maritisma* and *Vistrolepisma* nom. nov. from Australia. For comparison with Heterolepismatinae from other continents (all of them attributed so far to the genus *Heterolepisma* s. str.), see Table 5.

Character	*Lapidisma* gen. nov.	*Cactisma* gen. nov.	*Silvestrisma* gen. nov.	*Vistrolepisma* nom. nov.	* Maritisma *
Urotergite I chaetotaxy	3+3 combs	2+2 combs	2+2 or 3+3 combs	2+2 or 3+3 combs	3+3 combs
	A: 6	A: 2	A: 1–3	A: 1–3	A: 1
	B: 4–5	B: 1	B: 1–3	B: 1–2	B: 1
	C: 1 or 2	C: 0	C: 0–1	C: 0–1	C: 1
Urotergites II–VII chaetotaxy	3+3 combs	3+3 combs	3+3 combs	3+3 combs	3+3 combs
	A: 7–8	A: 3–5	A: 2–5	A: 1–6	A: 1–3
	B: 5	B: 2–3	B: 1–4	B: 1–4	B: 1–3
	C: 1–2	C: 1–2	C: 1–2	C: 1	C: 1
Urotergite VIII chaetotaxy	3+3 combs	2+2 combs	2+2 or 3+3 combs	2+2 combs	2+2 combs
	A: 8	A: 2	A: 3	A: 2–5	A: 1–2
	B: 5	C: 1–2	B:	C: 1	C: 1
	C: 1–2		C: 1–2		
Urotergite IX chaetotaxy	Without setae	Without setae or with few cilia	Variable. Bare or few infra-lateral setae	With infralateral groups of cilia	With 3+3 infralateral setulae
Urotergite X hind margin and shape	Subtrapezoidal short to subtriangular, slightly acute.	Truncate. Short trapezoidal shape	Relatively long, round, or sometimes approaching trapezoidal	Usually convex short	Slightly convex and very short
Scales on basal divisions of cerci	Some lanceolate long, narrow acute scales	Not detected, even with SEM	Absent or narrow lanceolate and acute	Lanceolate, narrow, and acute; triangular in some species	Absent
Urosternite I chaetotaxy	Without setae?	Without setae?	Variable (with or without a median comb)	Without setae	With one median comb
Urosternites II–VII chaetotaxy	With 1+1 single macrochaetae	With 1+1 single macrochaetae	Variable (1+1 macrochaetae or 1+1 combs)	Usually with 1+1 single macrochaetae	With 1+1 combs of 4–12 macrochaetae
Urosternite / Coxite VIII	1+1 single macrochaetae	With 2+2 macrochaetae	Variable (1+1 macrochaetae or 1+1 combs)	With 1+1 single macrochaetae	With 1–2 + 1–2 macrochaetae
Number of pairs of styli	4 pairs in both sexes	Only one pair	1–3 pairs; in some species, differences between sexes	3–6 pairs; in some species differences between sexes and between genetically similar populations	Only one pair
Scales on styli	Not visible in SEM images	Not detected with SEM	Absent	Triangular	Absent
Parameres	Large, ovoid, as long as the ninth coxite and with an apical division	No males collected	Small, conical, more or less long, smaller than the ninth coxite	Small, conical, more or less long, smaller than the ninth coxite	Relatively large, conical, almost as long as the ninth coxite

##### Etymology.

This generic name is a fusion of the words *lapidum* and *lepisma*, this latter used for most genera of silverfish belonging to the family Lepismatidae. *Lapidum* is the genitive plural form of *lapis*, a Latin word that means stone, and refers to the usual habitat of this insect, which is found associated with stones, usually under them. Thus, the literal translation of the word would be ‘the silverfish of stones’. The grammatical gender of this genus is neuter following the opinion of ICZN (2018) for all genera derived from *Lepisma*.

#### ﻿ Lapidisma paposanum sp. nov.

Taxon classificationAnimaliaZygentomaLepismatidae

C01ACF83-64C1-53AE-8CA0-E3740EBDB8FA

https://zoobank.org//77D018FD-4D45-4283-8776-623035A201F7

Figs 1, 2, 3, 4, 5, 6, 7, 8, 9

##### Type material.

***Holotype***: Chile • Antofagasta Region, Paposo, La Rinconada, under stones; 24°56.644'S, 70°29.70'W; 60 m a.s.l.; 01.IX.2023. Leg. A. Zúñiga. 1♀* mounted on slide [MNNC]. ***Paratypes***: Same collection data as for the holotype. Two specimens mounted on slide: 1♂* [MZUC], 1♂ [UCO], two specimens mounted on disc and gold coated: 1♂ [MZUC], 1♂ [MNNC] and two specimens preserved in ethanol 70%: 2♀ [UCO]. Chile • Antofagasta Region, Paposo, El Gaucho, under stones; 24°57.589'S, 70°28.489'W; 65 m a.s.l.; 29.IX.2024. Leg. A. Zúñiga. Four specimens, preserved in ethanol 70%: 1♂* [MNNC], 1♂/1♀ [MZUC], 1♂ [UCO].

##### Diagnosis.

As indicated for the genus. Infralateral combs with 6–8 macrochaetae (*L. annectens* has 5 macrochaetae on each infralateral comb). Ovipositor surpassing the apex of coxites IX by ~3.6× their length (~5× in *L. annectens*).

##### Description.

**Figure 1.**
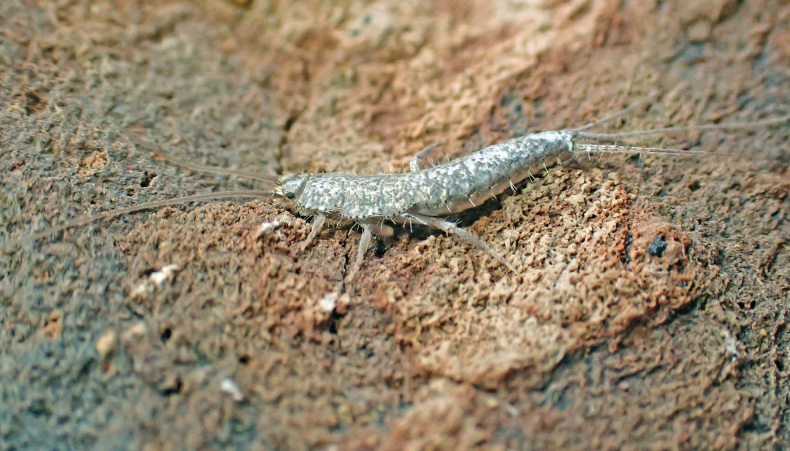
*Lapidisma paposanum* sp. nov., living specimen from La Rinconada (type locality) near Paposo (Atacama Desert, Chile), with the typical behaviour of clinging to the lifted stone Photo: RP.

**Figure 2.**
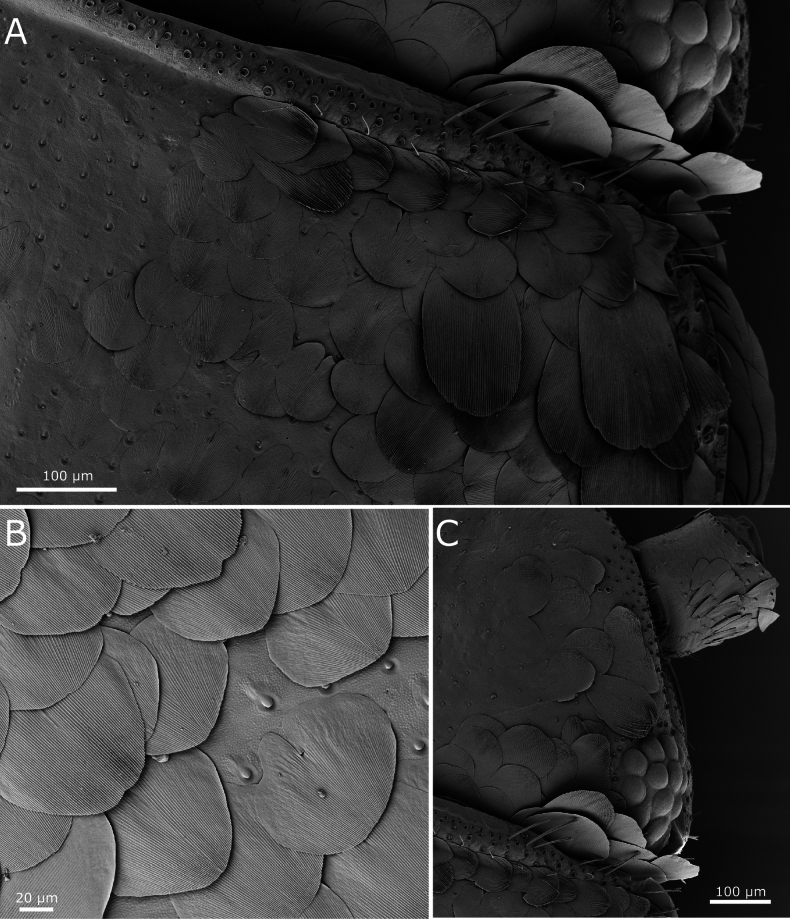
*Lapidisma paposanum* sp. nov., SEM images of scales. **A.** Scales on anterolateral margin of the pronotum; **B.** Scales on urotergite I; **C.** Scales on periocular region.

Habitus as in Fig. 1. Body length of holotype: 8 mm. Maximum body length observed in type series: 8.5 mm. Maximum preserved length of antennae in type specimens is 5 mm, but in live specimens antennae are as long as the body or slightly longer. Epidermal pigment present, brownish or brownish-violet, more intense on head, antennae (where it is uniform), legs (especially on tibiae, first tarsomere and apex of femora), lateral margins of dorsal plates, hind margins of urotergites, darker on the posterior segments and caudal filaments; ventrally, the pigment is more intense on the outer margins of coxites IX in both sexes and on the apex of parameres in males. Macrochaetae smooth, bifid, hyaline or yellowish. Body covered with orbicular scales (i.e., with lateral expansions surrounding the socket), their shapes are rounded or ovoid to subquadrangular (Fig. 2A, B); rounded ones are ~75 µm long, but some large subquadrangular can reach 170×100 µm; they show very dense parallel ribs, which are sometimes slightly convergent in the middle of the distal margin. Scales are also present on scape and pedicel, on the basal part of the maxillary palp, on legs (except tarsi) and on caudal filaments; the shape and size of these scales on appendages are modified compared to those covering the body, except for those on coxae.

**Figure 3.**
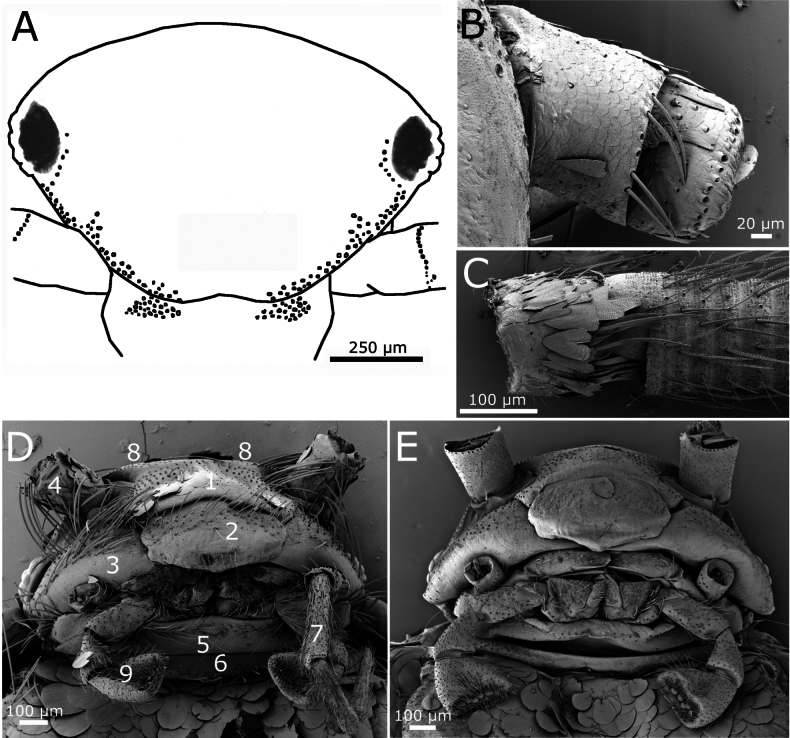
*Lapidisma paposanum* sp. nov. **A.** Schematic overview of the head (frontal view) showing frontal and clypeal chaetotaxy; **B.** Scapus and pedicel with some triangular scales; **C.** Pedicel with triangular scales and basal annuli of the flagellum with chaetic sensilla and trichobothria; **D.** Head in fronto-ventral view; 1: clypeus, showing transverse fringe of macrochaetae and small rounded scales, 2: labrum, with a proximal transverse fringe of macrochaetae and a short row of five median setae distally, 3: mandible, 4: scape with triangular scales, 5: mentum (labium), 6: prothoracic presternum, 7: antepenultimate article of maxillary palp with very small triangular scales on its basal part, 8: lateral fringes of frontal macrochaetae, median part of the frons without setae, 9: apical article of the labial palp; **E.** Head in fronto-ventral view, specimen cleaned by ultrasonic treatment.

Compound eyes with 12 ommatidia (Fig. 2C). Cephalic capsule with rounded scales, except on labrum. Frontal chaetotaxy extending from the periocular areas to the middle, where there is a median gap without setae (Fig. 3A). This marginal field of macrochaetae has two or three rows, including the periocular areas; only in the area close to the insertion of antennae there is a group of macrochaetae where additional macrochaetae can be observed. There are 1+1 lateral tufts of macrochaetae covering the clypeus; they can be clearly separated in the middle or relatively close together, without a wide median gap; the distal area of the clypeus bears two or three rows of rounded scales similar to those covering the head. Labrum with a continuous field of setae on its basal part and a row of five or six small setae of heterogeneous length in its distal part; the labrum is more or less folded backwards (Fig. 3B).

**Figure 4.**
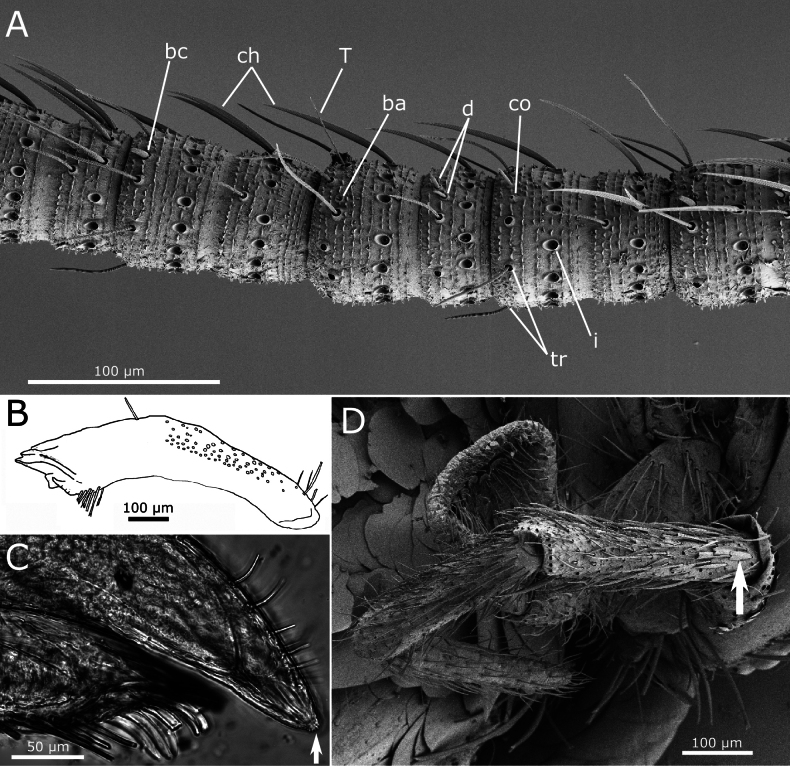
*Lapidisma paposanum* sp. nov. **A.** Different types of sensilla on the antennal flagellum; ba: basiconic sensillum type A, bc: basiconic sensillum type C, ch: chaetic sensilla, co: coelonocic sensillum, d: basiconic sensilla (diad), i: insertion of a chaetic sensillum, T: trichobothrium, tr: trichoid sensillum; **B.** Schematic drawing of the mandible; **C.** Micrography of the apex of galea and lacinia, with apex of the galea showing a small tubercle (arrow); **D.** Maxillary palp with some small triangular scales on the base of its antepenultimate article (one of them marked with an arrow).

Scape longer than pedicel, both with subtriangular scales, smaller than those covering the body (~50 × 20 µm), frequently showing a median indentation at their truncated apex, and in this case, they can be described as bifid (Fig. 3C, D). Antennal flagellum with chaetic, trichoid, and basiconic sensilla, as well as trichobothria. The latter are visible on all annuli, at least two per flagellomere in the basal area of the flagellum (Fig. 3D), more widely spaced in the distal part. Chaetic sensilla arranged in a row (ring) around each flagellomere, similar to setae of the body, but not bifid apically and slightly flattened. Trichoid sensilla present on most flagellomeres; similar to chaetic sensilla but thinner and shorter. Two types of basiconic sensilla visible in SEM images, attributed to types A and C described by Adel (1984); in addition, coeloconic sensilla have been detected (Fig. 4A).

**Figure 5.**
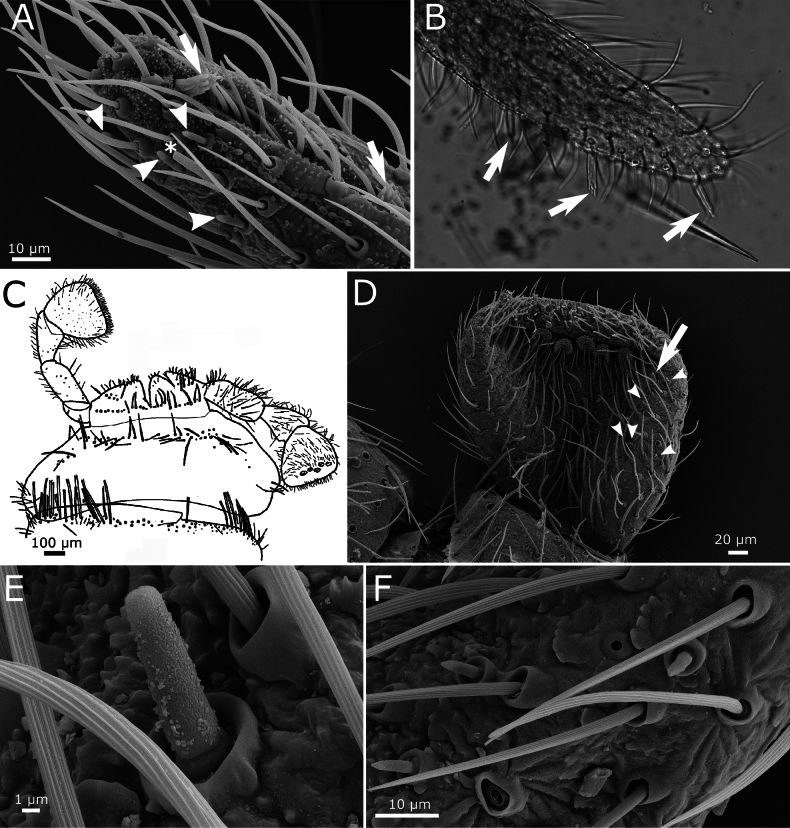
*Lapidisma paposanum* sp. nov. **A.** Apical part of distal article of the maxillary palp with styloconic sensilla (arrows), basiconic sensilla type B (arrowheads), and basiconic sensillum type C (asterisk); **B.** Micrograph of the distal article of the maxillary palp of the holotype with three styloconic sensilla (arrows); **C.** Schematic drawing of ventral overview of labium and presternum; **D.** Apical article of the labial palp with five papillae arranged in two very closely spaced rows; basiconic sensilla type B marked with arrowheads, basiconic sensillum type C marked with an arrow; **E.** Enlarged section of (**D**) with basiconic sensillum type C; **F.** Idem, with three basiconic sensilla type B.

Mandibles with an incisive and a molar area, with an outer field of ~75–90 macrochaetae; below the molar area there is a small tuft of seven short macrochaetae (Fig. 4B). Galea with a small apical tubercle (Fig. 4C). Lacinia with ~7 lamellate processes and a row of six setae, at least one of them apically bifid. Maxillary palp with its apical article ~4.3–5.4× longer than wide and shorter or longer than the penultimate (ratio length of the last article / length of the penultimate ~0.75–1.27). In the basal part of the palp, the second article has some bifid setae and several subtriangular scales, very small (~30 µm long) and narrow, with truncated apical margin (Fig. 4D). The apical article has a longitudinal row of three styloconic sensilla with a very slender cylindrical style and five or six apical cones (Fig. 5A, B), a distal basiconic sensillum type C and at least four basiconic sensilla type B (Fig. 5A).

Labium as in Fig. 5C, with mentum clearly wider than long, the labial palp with a widened apical article (maximum width 1.1× wider than its length and 1.6× the maximum width of the penultimate article) which bears five papillae arranged in a jagged line; they could be described as arranged in two lines of 3+2 papillae, but these rows are very close together (Fig. 5C, D). In one specimen, an apical article has only four papillae (3+1). A single basiconic sensillum type C and four or five basiconic sensilla type B in the outer lateral part of the apical article (Fig. 5D−F).

**Figure 6.**
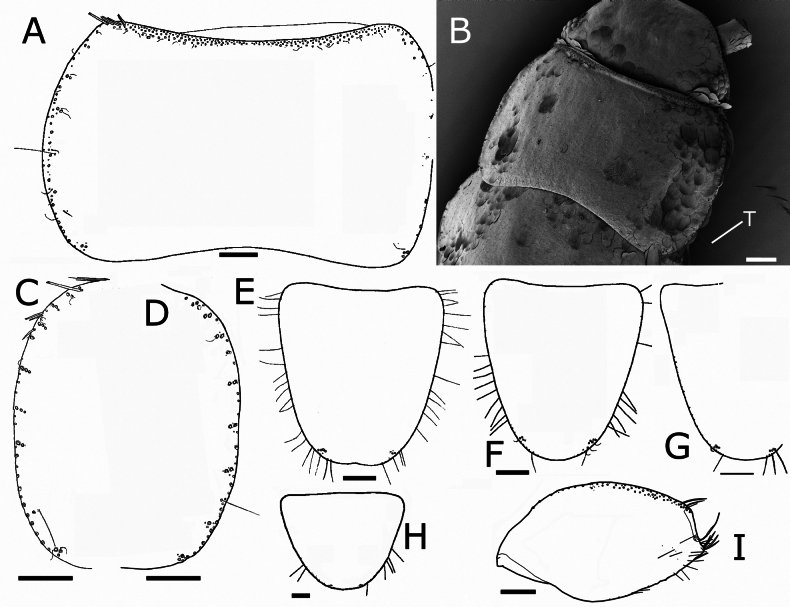
*Lapidisma paposanum* sp. nov., thorax. **A.** Pronotum with chaetotaxy of the anterior margin (collar), left lateral margin (one trichobothrium is preserved) and the posterior margin lacking setae; **B.**SEM image of pronotum, showing the position of a posterior trichobothrium (T); **C.** Mesonotum, left margin; **D.** Metanotum, right margin (insertions of most setae and position of the anterior trichobothrium visible); **E.** Prosternum of the holotype, on the posterior apical part combs of macrochaetae are visible; **F.** Prosternum of another specimen; **G.** Mesosternum, right margin and posterior apical part with combs of macrochaetae; **H.** Metasternum with combs of macrochaetae barely visible on the posterior margin. **I..** Metacoxa with chaetotaxy. Scale bars: 0.2 mm (**A, C–I**); 0.1 mm (**B**).

Pronotum with a setal collar consisting of three or four rows of macrochaetae that are gradually reduced to a single row in the middle and at the anterolateral corners (Fig. 6A). The three thoracic nota with several marginal macrochaetae on their lateral margins and lacking setae on the posterior margins. Apart from marginal macrochaetae, some submarginal macrochaetae on lateral margins, arranged in small combs. On the pronotum, three combs of two macrochaetae on the anterior part of the lateral margin, and another one on the posterolateral corner bearing two macrochaetae; this latter comb cannot be considered as a posterior comb. Between the anterior combs and the posterolateral one, there is a section of the lateral margin with only some isolated submarginal macrochaetae; several of these (two to five on each side) can be interpreted as combs formed by one macrochaeta. Some small setae and curved trichoid sensilla are associated with these combs. Trichobothrial areas difficult to see because trichobothrial hairs are mostly detached, but one trichobothrium confirmed at ~0.55 of the length of the lateral side, which can be interpreted as an anterior trichobothrial area (Fig. 6A); in other specimen a trichobothrium was detected in a more posterior position, at ~0.64 the length of the notum, corresponding to a posterior trichobothrial area (Fig. 6B).

Mesonotum with 9+9 combs of macrochaetae, those on the anterior part of the lateral margin with two or three macrochaetae, and the most posterior ones with one or two macrochaetae (the one at the posterolateral edge has two). One trichobothrium, apparently not associated with a comb, on the lateral margin at ~0.6 of its length, and another one of another specimen at a more posterior position, associated with the penultimate lateral comb, at ~0.8 of the length of the mesonotum (Fig. 6C).

The metanotum (Fig. 6D) has 10+10 combs of one or three macrochaetae, the number of combs with three macrochaetae is lower than on the mesonotum and these are present in the anterior part of the lateral margin. A lateral trichobothrium on the posterior part of the lateral margin at ~0.8 of the length of the metanotum; the position of the anterior trichobothrium has not been clearly discerned.

Presternum of prothorax with two transverse rows of macrochaetae. Prosternum subquadrangular to cordiform, slightly longer than its maximum width (ratio L/W ~1.1), with 1+1 antedistal combs of two or three macrochaetae, its hind margin truncate or slightly rounded (Fig. 6E, F); lateral margins with several thin and long setae. Mesosternum with a similar ratio L/W and shape; the 1+1 antedistal combs with three or four macrochaetae, and with a lower number of thin and long setae in its lateral margin (Fig. 6G). Metasternum wider than long; ratio L/W ~0.81; 1+1 combs of two or three macrochaetae; thin and long setae less abundant and limited to the posterior part of the lateral margin. The hind margin of this sternite widely rounded (Fig. 6H).

Anterior margin of coxae with a dense row of macrochaetae, usually arranged in oblique combs of two or three macrochaetae; the posterior margin with one row of few macrochaetae, mainly inserted on the distal part of the article (Fig. 6I).

**Figure 7.**
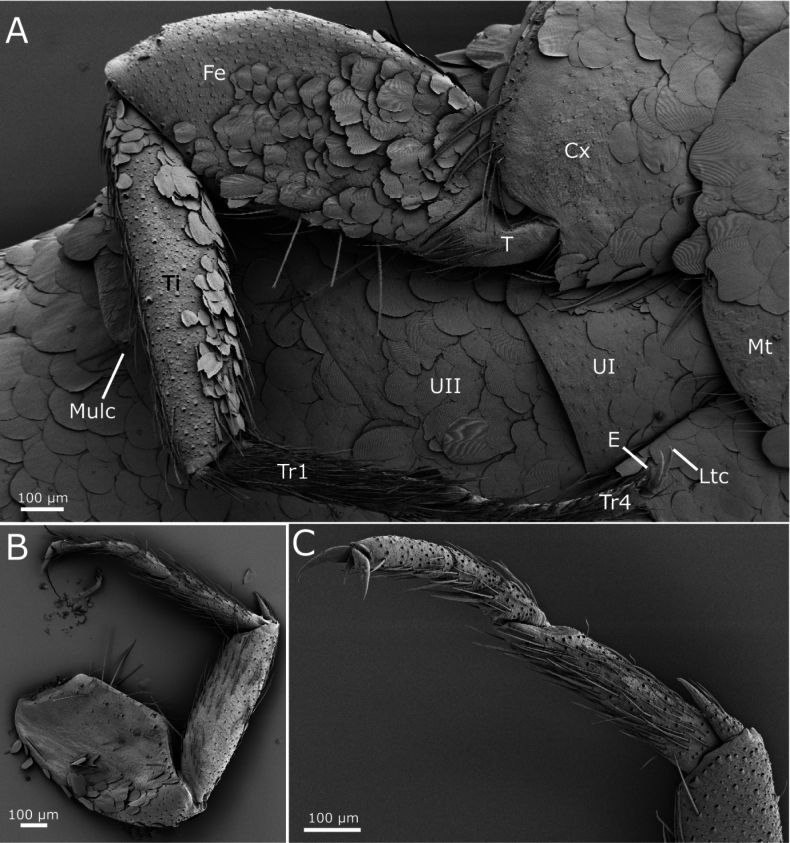
*Lapidisma paposanum* sp. nov., SEM images of legs. **A.** Ventral view of the third leg showing scales with special shape on femur and tibia; **B.** Outer side of the third leg (the side observed dorsally, except for coxa and trochanter), scales with special shape can be observed on the femur; **C.** Ventral view on apical part of protibia with tibial spur and tarsus with pretarsal claws. Abbreviations: Cx: coxa, E: empodial claw, Fe: femur, Ltc: lateral claws, Mt: metasternum, Mulc: lateral comb of urosternite III consisting of two macrochaetae of different length, T: trochanter, Ti: tibia, Tr1: first tarsomere, Tr4: fourth tarsomere, UI/II: first/second abdominal sternite (urosternite I/II).

Femora covered with scales, more numerous on the inner (ventral) side, where they are oval to rounded, smaller than those covering the body (~60–70 µm long), with the apical margin truncated, with some shallow indentations (Fig. 7A); on the outer (dorsal) side, scales are limited to the antero-apical area and their shape is more heterogeneous, some of them are narrower and/or are bifurcated on their apical margin (Fig. 7B). The inner side of tibiae is covered with rounded to oval scales, similar in shape to those of femora, but smaller (most of them are <50 µm long; see Fig. 7A); the outer side of this article is apparently devoid of scales, only covered with setae (Fig. 7B). Most setae of femora and tibiae detached and only their insertions are visible; some insertions are larger, suggesting that they correspond to small macrochaetae. When preserved, macrochaetae are shorter than the diameter of tibiae; on protibiae one macrochaeta is present on the median part of the anterior (dorsal) margin and three are visible on the hind margin (one basal, one median and one subapical; see Fig. 7C); on metatibiae two insertions are visible on the anterior margin and the number of macrochaetae is higher at the posterior margin, but variable in the available specimens; in SEM images insertions of setae with an intermediate size are visible, smaller than insertions of macrochaetae and larger than those of normal setae (Fig. 7A). Protibiae ~3.4–3.9× longer than wide; metatibiae ~4.1–4.7× longer than wide and ~1.25× longer than protibiae. Tarsomeres lacking scales; tarsomere 1 of the third leg ~0.57× the length of the metatibiae, 2.25× longer than the tarsomere 2, ~4× longer than the tarsomere 3 and 2.2× longer than the fourth tarsomere; these proportions in the first leg are 0.55, 2.4, 3, and 2, respectively. Pretarsal claws smooth, as in Fig. 7C; the medial empodial claw ~1/2 the length of lateral claws.

**Figure 8.**
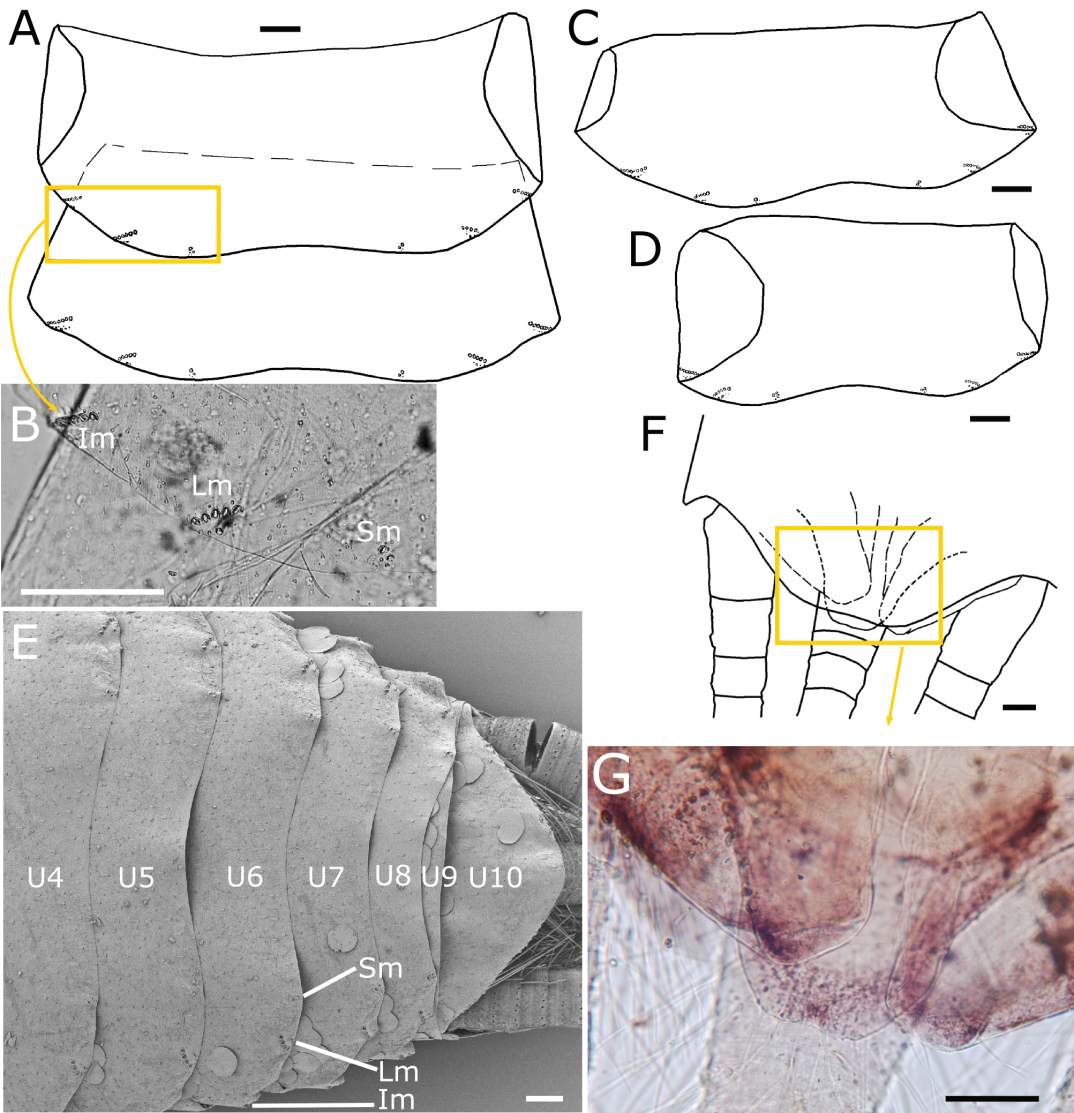
*Lapidisma paposanum* sp. nov., abdomen, dorsal. **A.** Urotergites I and II with insertions of macrochaetae (combs); **B.** Micrograph showing in detail the combs on urotergite I marked in A; **C.** Urotergite V; **D.** Urotergite VI; **E.**SEM image of the posterior urotergites IV–X (marked as U4–U10), Lm: macrochaetae of lateral comb, Sm: macrochaetae of submedian comb; **F.** Hind margin of urotergite X and position of epiproct and paraprocts; **G.** Micrograph showing the area marked in **F.** Scale bars: 0.1 mm. Abbreviations: Im: macrochaetae of infralateral comb, Lm: macrochaetae of lateral comb, Sm: macrochaetae of submedian comb.

Urotergites I−VIII with 3+3 combs of macrochaetae. The infralateral combs bear 6–8 macrochaetae and the lateral combs 3–5. At a submedian position there are one or two macrochaetae on each side (Fig. 8A, E) which are interpreted as reduced submedian combs. When the submedian comb consists of two macrochaetae, they are either of equal size or the more posterior one is smaller (Fig. 8A, D, E). In several cases (for example, on the urotergite VII of the holotype), the smaller macrochaeta is absent (Fig. 8C) or even the entire comb disappears (for example, the holotype has an asymmetric chaetotaxy on urotergite VI, with 2+3 combs, lacking the submedian one on one side and, on the other side, there is only one macrochaeta). Some small setae are inserted posteriorly to the combs, close to the hind margin of the tergite.

Urotergite IX reduced in length and lacking setae. Urotergite X short, with hind margin convex, almost straight or rounded, in some specimens forming an ill-defined obtuse subtriangular area in the middle (Fig. 8E) and in some others slightly concave (Fig. 8F); it has only some setae on their lateral margins but lacks combs of macrochaetae.

**Figure 9.**
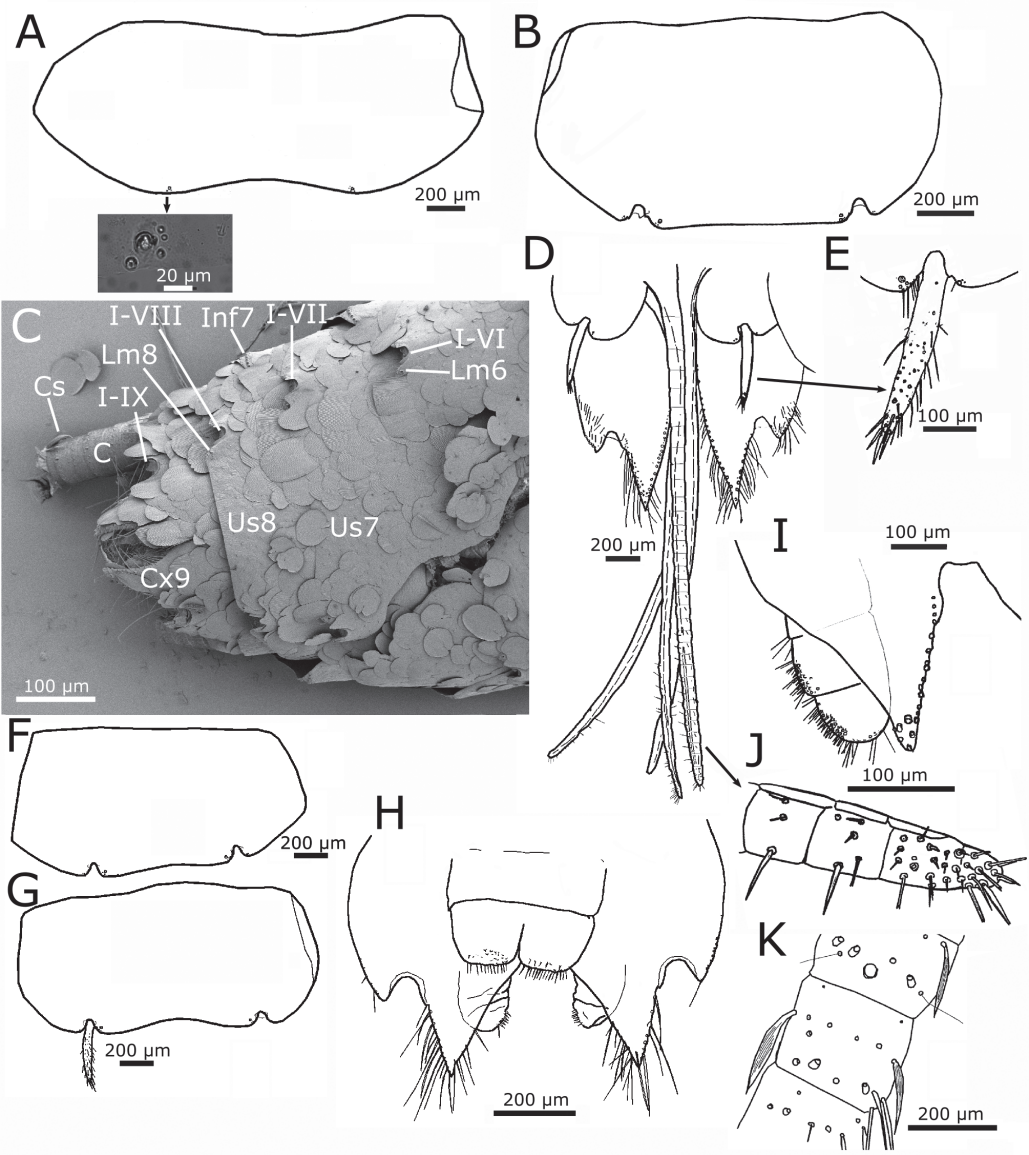
*Lapidisma paposanum* sp. nov., abdomen, ventral. **A.** Urosternite IV with insertions of lateral macrochaetae, the micrograph showing the details of one insertion, surrounded by additional insertions of smaller setae; **B.** Urosternite VIII with insertions of lateral macrochaetae and styli on the posterior margin; **C.**SEM image of the posterior abdominal segments of a male specimen, the straight margin of urosternite VIII is clearly visible, while that of the urosternite VII is mostly covered by scales; **D.** Coxites VIII and IX of the female holotype with ovipositor, styli IX are detached and only their insertions are visible; **E.** Detail of the left stylus on coxite VIII; **F.** Urosternite VI with insertions of styli; **G.** Urosternite VII with a single stylus preserved; **H.** Coxites IX of a male showing the penis and parameres; **I.** Detail of paramere next to the inner process of the coxite IX of another male specimen; **J.** Apex of gonapophysis VIII; **K.** Detail of some cercal divisions showing insertions of diverse types of setae, trichobothria and narrow lanceolate scales. Abbreviations: C: cercus, Cs: cercal scale, Cx9: coxite IX (parameres and penis hardly visible below its inner margin), I: insertions of styli, Inf7: infralateral comb of macrochaetae of urotergite VII (part of the dorsal tergite bent ventrally), Lm6/8: lateral macrochaetae on posterior margin of urosternites VI/VIII, Us7/8: discs of urosternites VII/VIII.

Urosternite I devoid of setae, its median part is broken in the holotype (but visible in another specimen; see Fig. 7A). Urosternites II−VIII with 1+1 isolated lateral macrochaetae (Figs 7A, 9A). Each of these macrochaetae is accompanied by some smaller setae; frequently, one or two of these can be identified as trichoid sensilla surrounding the larger macrochaetae posteriorly. In males, the urosternites VIII entire (not divided into two lateral coxites), with a straight hind margin between the styli (Fig. 9B, C); in females, each coxite VIII has, apart from the isolated macrochaeta inserted on the hind margin outwards to the stylus, another macrochaeta inserted inwards, accompanied by two smaller setae (Fig. 9D).

Four pairs of abdominal styli in both sexes, inserted on segments VI−IX (Fig. 9). Coxites IX of males as in Fig. 9H, I, with the inner process ~1.15× longer than wide at its base and 4× longer than the outer process. In females, the inner process of the ninth coxite is ~1.4–1.5× longer than wide at its base and 3.5× longer than the outer process (Fig. 9D). The margins of the processes of coxites IX are covered by numerous long and thin setae. Males bear ovoid parameres, divided into a basal portion and an apical segment, this one bearing two small groups of glandular setae (Fig. 9I); the size of parameres is small to medium, not reaching the apex of inner process of coxite IX (the larger ones only slightly shorter in larger specimens, as in Fig. 9I, some others only with a length ~1/2 the length of coxite IX, as in Fig. 9H). The ovipositor has ~34 or 35 divisions and surpasses the apex of coxites IX by ~3.6× their length (Fig. 9D). Apex of gonapophyses VIII as in Fig. 9J. Epiproct well developed and pigmented, covering the base of the paracercus and almost as long or even longer than the urotergite X, when this tergite has a straight or slightly concave hind margin (Fig. 8F, G), flanked by two similarly large paraprocts. Terminal filaments pigmented, with at least one ring of macrochaetae on each division; in addition to chaetic and trichoid sensilla, trichobothria, and narrow lanceolate scales with acute or slightly truncated apex (Fig. 9K; one can be seen in Fig. 9C).

##### Habitat and behaviour.

**Figure 10.**
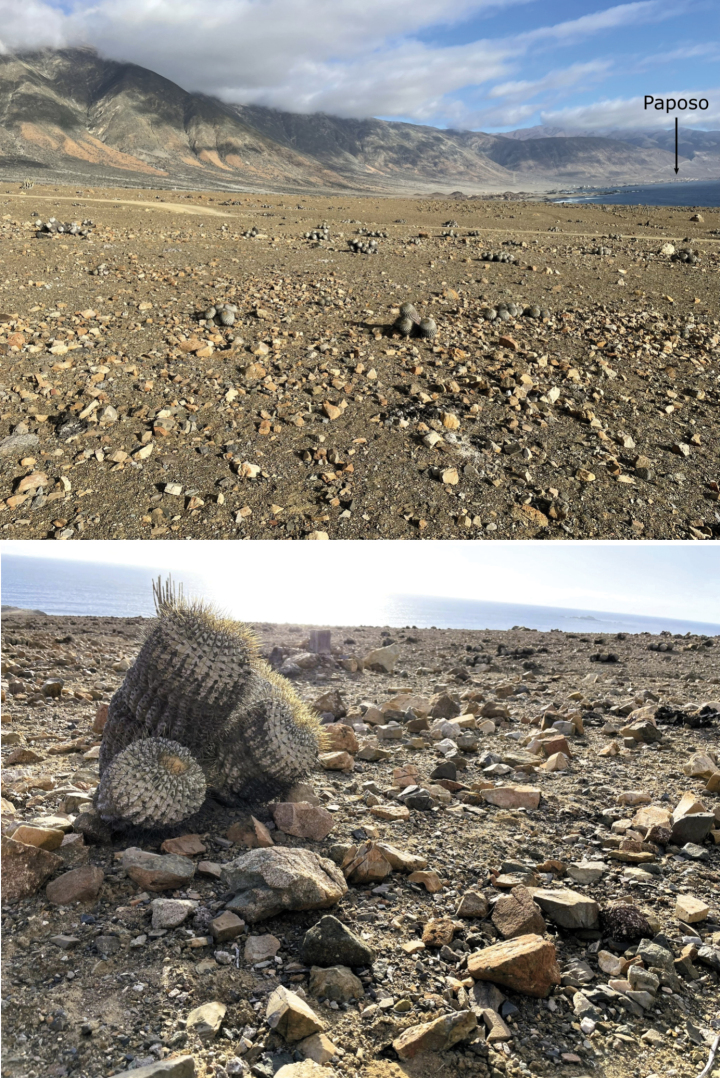
Habitat typical of *Lapidisma paposanum* sp. nov. in La Rinconada, Paposo (Atacama Desert, Chile), specimens were collected under the medium sized rocks.

The habitat is on a coastal terrace close to the beach dominated by cacti of the species *Copiapoa gigantea* Backeb. The sandy clay soil is covered by many medium to small stones (Fig. 10). Scarce perennial vegetation is supported by the strong fogs typical of the area (camanchaca), in addition the area receives irregular rainfall, occasionally resulting in a short-lived herbaceous layer of annuals. *Lapidisma paposanum* sp. nov. has been found under stones, clinging like *Thermobia* to the raised stones (Fig. 1).

##### Etymology.

The specific name refers to the village of Paposo, where the new silverfish species has been found.

#### ﻿ Cactisma gen. nov.

Taxon classificationAnimaliaZygentomaLepismatidae

EE24BF08-82D7-57DF-842F-AAF7CA495871

https://zoobank.org/51FF634A-64E1-45FE-A258-B4782731A736

##### Type species.

*Cactisma camanchaca* sp. nov.

##### Diagnosis.

Medium-sized silverfish (adults reaching 6.5–8.5 mm). Body shape slender, fusiform. Antennae as long or slightly shorter than body; caudal filaments almost as long as body length. Epidermal pigment scarce, dorsal scales pigmented. Scales covering the body dorsally and ventrally (including coxae) rounded to oval, orbicular, with more or less developed process surrounding sockets, multi-radiate and of diverse sizes. Scales absent on clypeus, labrum, antennae, maxillary and labial palps, abdominal styli, ovipositor, and caudal filaments. Scales of femora and tibiae with modified shape; smaller, subtriangular, narrow, with apical margin truncate or bifid, with a median indentation. Macrochaetae smooth, apically bifurcate. Compound eyes with 12 ommatidia, sometimes only 10 or 11 ommatidia developed. Frontal chaetotaxy concentrated on lateral parts, with a wide gap in the middle showing only one row of setae. Clypeus almost vertical, with a continuous transverse field of macrochaetae. Labrum with several irregularly arranged bifid setae. Antennae with trichobothria and also with chaetic, trichoid, and basiconic sensilla. Maxillary palps with the apical article bearing two or three styloconic sensilla, several basiconic sensilla type B and a single basiconic sensillum type C. Apical article of the labial palp with five papillae arranged in an oval shape, outer lateral part with several basiconic sensilla type B and a single basiconic sensillum type C. Anterior margin of pronotum with setal collar. Lateral margins of thoracic nota with several macrochaetae, most of them isolated, in some places forming a small comb of two macrochaetae; there are 1+1 posterior combs of two macrochaetae in a very lateral position, leaving the posterior margin bare. Trichobothrial areas open (Type 1 according to Mendes’ classification). Thoracic sternites parabolic, with convex or slightly truncated hind margins. Coxae with rows of macrochaetae on their anterior margin that are arranged in indistinct combs; the posterior margins with a single row of few long and thin macrochaetae. Tarsi consisting of four articles; pretarsus with two smooth claws and a medial empodial claw. Urotergites I−VIII with 3+3 combs of macrochaetae; the submedian combs sometimes consisting of only one macrochaeta. Urotergite IX without setae. Urotergite X short trapezoidal, with straight hind margin, several setae on its lateral margins and posterolateral edges; lacking clear combs. Urosternite I devoid of setae. Urosternites II−VII with 1+1 single macrochaetae; coxite VIII of females with one or two macrochaetae. One pair of abdominal styli. Females with a slender ovipositor of the primary type. Male unknown.

##### Remarks.

This new genus is different to *Lapidisma* gen. nov. from Chile and to the other genera of Heterolepismatinae by the characters listed in Tables 2, 3. It is clearly different to Australian *Heterolepisma* due to the presence of bifid macrochaetae on labrum (only small acute setae in Australian taxa), the absence of posterior combs of thoracic nota (present in Australian species), shorter urotergite X and different shape of scales covering appendages; to *Maritisma* due to the same differences except for the shape of urotergite X, and additional characters such as the different urosternal chaetotaxy and labial palp characters. *Cactisma* gen. nov. is different to *Vistrolepisma* (previously *Visma*) because of the absence of posterior combs on thoracic nota, different shape of thoracic sternites (widely truncate in *Vistrolepisma*), shorter urotergite X and lower number of abdominal styli. Differences to *Lapidisma* gen. nov. include different shape of scales of appendages, lower number of macrochaetae on infralateral urotergal combs, and lower number of pairs of styli. Problems arise when comparing *Cactisma* gen. nov. with some incompletely described species of *Heterolepisma* from regions other than Australia, as information on distribution and shape of scales on appendages or chaetotaxy of labrum is missing. Considering the characters available, *Cactisma* gen. nov. is different to other previously described species assigned to *Heterolepisma* due to the absence of macrochaetae on the hind margin of thoracic nota and shorter urotergite X. Apart from *Lapidisma* gen. nov. (and *H. annectens*, now assigned to *Lapidisma*), the only species sharing both characters, are the *Heterolepisma* described from Argentina, i.e., *H. andinum* and the type species of the genus, *H. pampeanum*. A specimen assigned to *H. andinum* was available for our study and differences concerning the shape of scales in appendages (lanceolate scales in legs, for example) and the frontal chaetotaxy indicate that the Argentine species indeed belong to a different genus, so *Heterolepisma* s. str. corresponds only to Argentine species (and, perhaps, to other species of this genus described from other geographic areas, but not from Chile so far); see Tables 4, 5, and additional comments in the Discussion section.

**Table 4.** Comparison of cephalic morphological characteristics between the new genera from Chile and the non-Australian species of *Heterolepisma*. Characters observed in *H. andinum* from Argentina are included in a separate column. Remaining species of *Heterolepisma* are very heterogeneous and their original descriptions often lack characters useful for comparison (see notes in the corresponding column).

Character	*Lapidisma* gen. nov.	*Cactisma* gen. nov.	*Heterolepisma andinum*	Remaining *Heterolepisma* (excluding Australian species)
Median gap on frontal fringe	Yes	Yes	No	Yes, but not clearly described for several species. The description of *H. pampeanum* suggests the absence of gap
Clypeal scales	Present, rounded (detected with SEM)	Not detected (SEM used)	Not detected	Not detected in most species
Bifid macrochae-tae on labrum	Yes	Yes	Yes	Not described in most species, apparently present in *H. bisetosum*
Scales on scapus	Wide truncate, subtriangular	Not detected (SEM used)	Lanceolate with an apical	Not described in most species
Scales on pedicel	Triangular, truncate or even bifid	Not detected, (SEM used)	Not detected	Not described in any species
Styloconic sensilla on maxillary palp	3, slender styles with several short cones	3, slender styles with several short cones	3, slender styles with several short cones	When described, the sensilla are similar to those of Australian species, short styles with several long cones (flower-shaped in *H. bisetosum*, *H. japonicum*, *H. serranoi* and *H. tonga*), less complex in females
Scales on maxillary palp	Narrow lanceolate or sub- triangular and truncate, on basal article	Not detected, (SEM used)	Not detected	Not described in any species
Papillae on labial palp	5 compact, forming two rows. Apical article wide.	5 compact, forming an oval shape. Apical article narrower.	Not described and not visible in the available specimen	When described, 5 compact (3+2 in two rows). Shape variable
Macrochaetae on posterior margins of thoracic nota	0	0	0	*H. annectens* lacks these macrochaetae. *H. pampeanum* also lacks posterolateral macrochaetae. The remaining species bear 1+1 or 2+2 macrochaetae
Scales on femora and tibiae	Wide, rounded; absent on outer side of tibiae	Wide, subtriangular (narrower than in *Lapidisma*)	Lancet shaped, with apical indentation or acute	In some species lancet-shaped (usually acute, without apical indentation), observed in *H. bisetosum*, *H. horni*, *H. mossambicense*, *H. serranoi* and *H. tonga*. Not described for some species
Thoracic sternites	Parabolic, with convex to slightly truncate hind margin	Parabolic, with convex hind margin	Parabolic, with convex hind margin	Cordiform with convex hind margin in most species, only in some the metasternum is truncated posteriorly. *H. pampeanum* is the most similar to the new genera and to *H. andinum*. Not described in some species

**Table 5.** Comparison of abdominal morphological characteristics between the new genera from Chile and the non-Australian species of *Heterolepisma*. Characters observed in *H. andinum* from Argentina are included in a separate column. Remaining species of *Heterolepisma* are very heterogeneous and their original descriptions often lack characters useful for comparison (see notes in the corresponding column). *, according to the original description and/or with the key of Stach (1933).

Character	*Lapidisma* gen. nov.	*Cactisma* gen. nov.	*Heterolepisma andinum*	Remaining *Heterolepisma* (excluding Australian species)
Urotergite I chaetotaxy	3+3 combs	2+2 combs	3+3 combs	For some species 2+2 or 3+3 combs described, for other species data missing
	A:6	A: 2	A: 4	
	B: 4–5	B: 1	B: 4	
	C: 1 or 2	C: 0	C: 1 or 2	
Urotergites II–VII chaetotaxy	3+3 combs	3+3 combs	3+3 combs	3+3 combs with variable number of macrochaetae, usually less than in *Lapidisma* gen. nov.
	A: 7–8	A: 3–5	A: 4–5	
	B: 5	B: 2–3	B: 4	
	C: 1–2	C: 1–2	C: 1–2	
Urotergite VIII chaetotaxy	3+3 combs	2+2 combs	2+2 combs	Some species with 2+2 combs, some others with 3+3 combs.
	A: 8	A: 2		
	B: 5,	C: 1–2		
	C: 1–2			
Urotergite IX chaetotaxy	Without setae	Without setae or with few cilia	Without setae?	Some species lack setae and some others with 1+1 infralateral with one or few reduced setae
Urotergite X hind margin and shape	Subtrapezoidal short to subtriangular, slightly acute.	Truncate. Short trapezoidal shape	Short and slightly convex.	Long trapezoidal in most species, straight or slightly convex hind margin
Scales on basal divisions of cerci	Some lanceolate long, narrow, and acute scales	Not detected, even with SEM	Not detected	Not observed in most species
Urosternite I chaetotaxy	Without setae?	Without setae?	? (damaged)	Several species with a median comb, but without setae in some others. Inadequately reported in some species, including the type species *H. pampeanum*
Urosternites II–VII chaetotaxy	With 1+1 single macrochaetae	With 1+1 single macrochaetae	1+1 combs with several (3 or more macro-chaetae)*	For some species 1+1 single macrochaetae, for others 1+1 combs (probably including *H. pampeanum*) described, for other species data missing
Urosternite / Coxite VIII	1+1 single macrochaetae	With 2+2 macrochaetae	1+1 combs with 3 macrochaetae	Most species with 1+1 or 2+2 macrochaetae
Number of pairs of styli	4 pairs in both sexes	Only one pair	Only one pair	Variable. Usually the number is 1–3 pairs of styli, with sexual dimorphism in some species
Scales on styli	Not visible in SEM images	Not detected in SEM images	Not observed	Not observed
Parameres	Large, ovoid, as long as the coxite IX and with an apical division	No males collected	Males not observed	Usually very small, only large ovoid in *H. annectens* and *H. insulare*

##### Etymology.

This generic name is a fusion of the words ‘cactus’ and ‘lepisma’, the latter used for most genera of Lepismatidae. Cactus derives through Latin from the ancient Greek word κάκτος used for an indetermined spiny plant and currently used for designating plants belonging to the family Cactaceae; decaying specimens of these plants provide the habitat where this new silverfish has been found. The grammatical gender of this genus is neuter.

#### ﻿ Cactisma camanchaca sp. nov.

Taxon classificationAnimaliaZygentomaLepismatidae

6C448F75-0B2A-5936-B7B7-A3559EDC7CCC

https://zoobank.org/E232770E-672A-4FD7-BD29-FDAC0A1E7FFD

**Figure 11.**
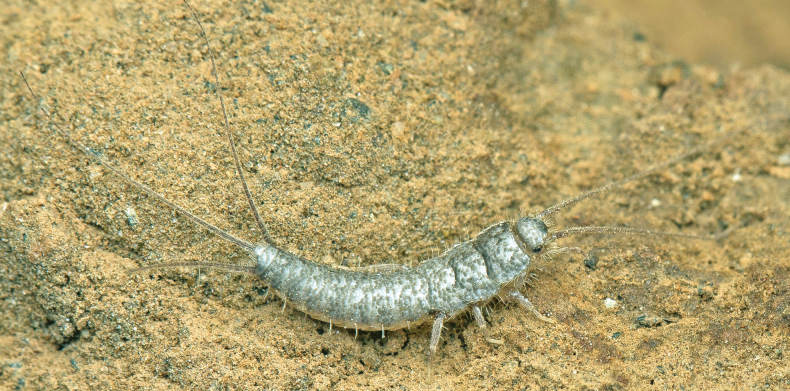
*Cactisma camanchaca* sp. nov., living specimen from the type locality at Cuesta el Cobre, Atacama Desert, Chile. (Photo: P. Pinto).

**Figure 12.**
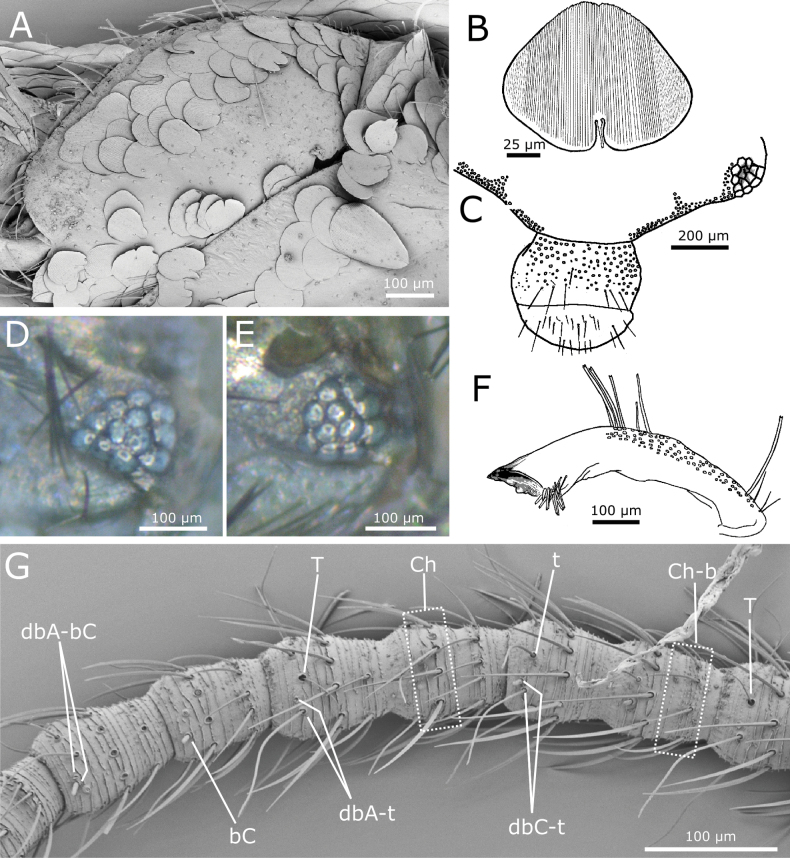
*Cactisma camanchaca* sp. nov. **A.** Typical rounded scales covering the body (procoxa and part of the prosternum); **B.** Detail of an orbicular dorsal scale; **C.** Overview of chaetotaxy of frons, clypeus and labrum; most setae represented by their insertions only; **D.** Compound eye showing 12 ommatidia; **E.** Idem, another specimen with 11 ommatidia; **F.** Mandible; **G.** Part of the antennal flagellum. Abbreviation: bC: basiconic sensillum type C, Ch: row of chaetic sensilla, Ch-b: second row of chaetic sensilla, present in a more basal position on some annuli, dbA-bC: diad consisting of a basiconic sensillum type A and a basiconic sensillum type C, dbA-t: diad consisting of a basiconic sensillum type A and a trichoid sensillum, dbC-t: diad consisting of a basiconic sensillum type C and a trichoid sensillum, t: trichoid sensillum, T: trichobothrium.

**Figure 13.**
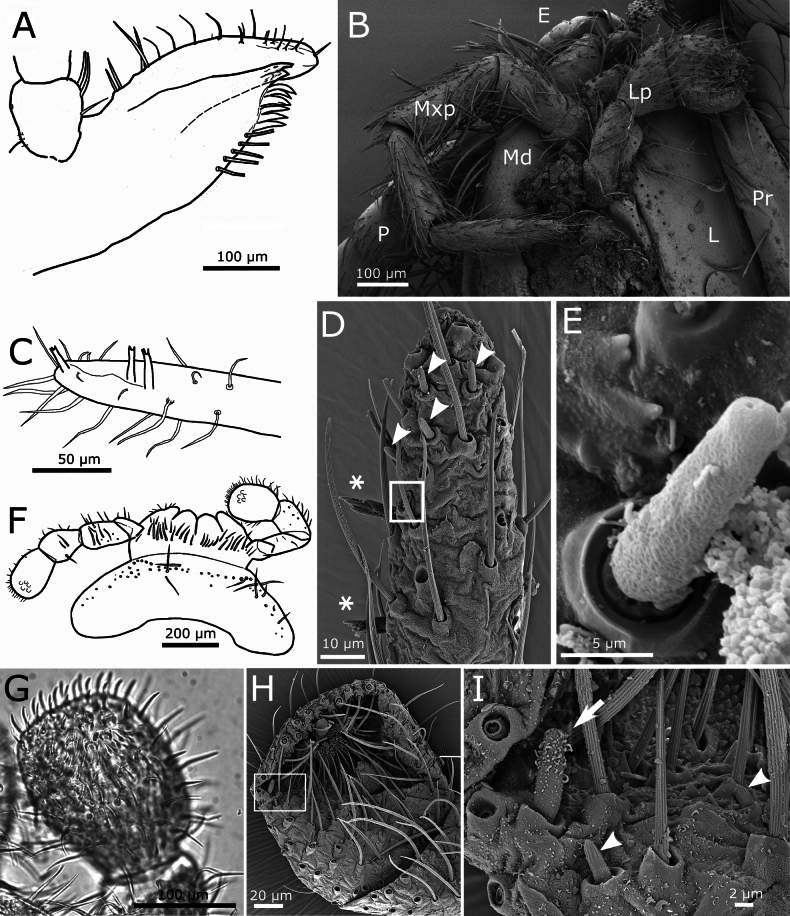
*Cactisma camanchaca* sp. nov. **A.** Apex of lacinia and galea; **B.** Ventral side of the head (right part); **C.** Apical part of the distal article of the maxillary palp with three styloconic sensilla; **D.** Idem, detail with two styloconic sensilla (asterisks), four basiconic sensilla type B (arrowheads), and the socket of a basiconic sensillum type C (box); **E.** Intact basiconic sensillum from distal article of the maxillary palp of another specimen, showing a single apical pore; **F.** Labium with palps; **G.** Micrograph of the apical article of the labial palp of the holotype, showing the five sensory papillae arranged in an oval shape; **H.** Distal article of labial palp, area with basiconic sensilla marked by a white box; **I.** Detail of (**H**) with single basiconic sensillum type C (arrowhead) and two basiconic sensilla type B (arrowheads). Abbreviations: E: eye, Lp: labial palp, Md: mandible, Mxp: maxillary palp, L: labium (mentum), P: antennal pedicel, Pr: presternum.

**Figure 14.**
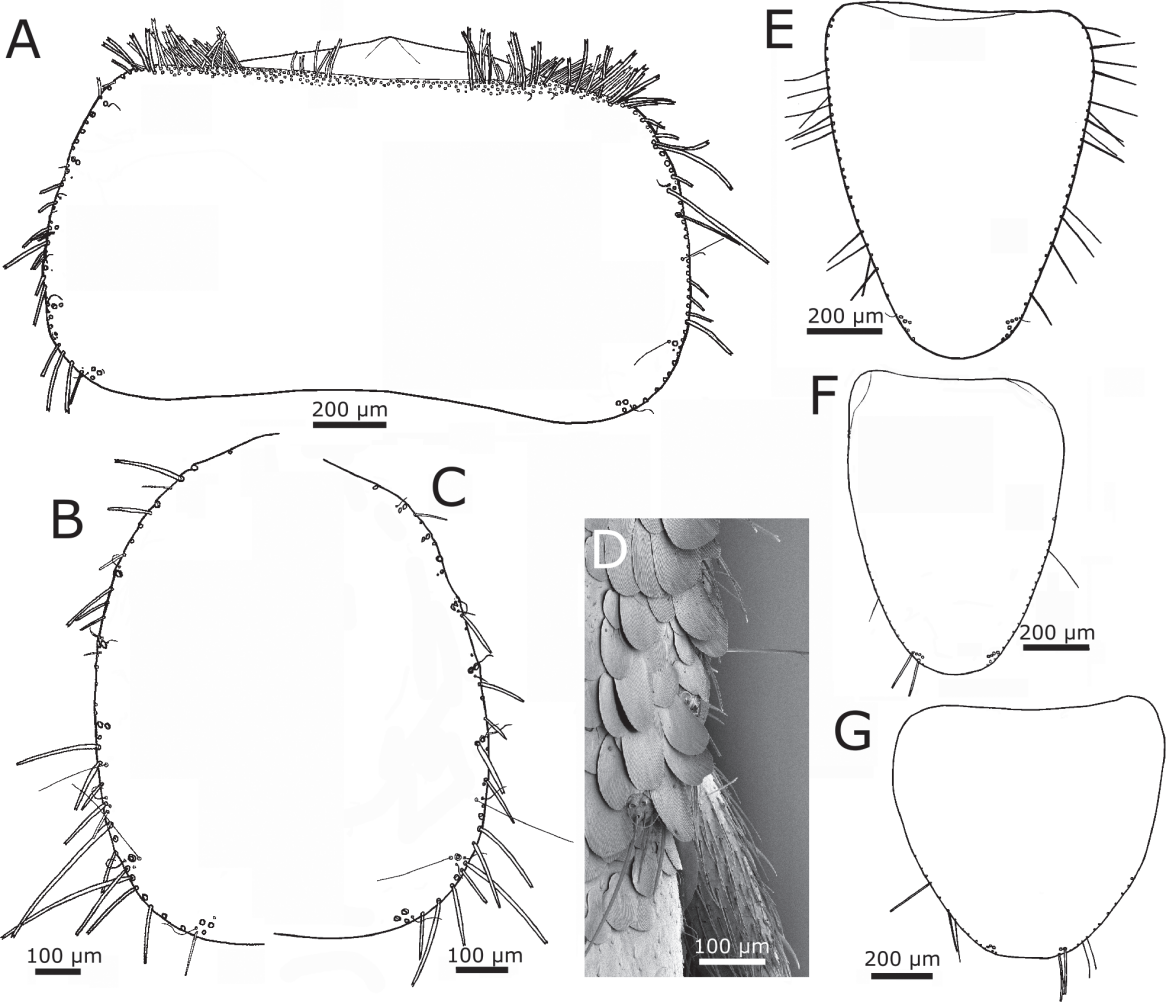
*Cactisma camanchaca* sp. nov. **A.** Pronotum with position of trichobothria visible on the right side; **B.** Mesonotum, left margin; **C.** Metanotum, right margin; with anterior and posterior trichobothria visible; **D.**SEM image of the posterior lateral comb of the metanotum; **E.** Prosternum; **F.** Mesosternum; **G.** Metasternum.

**Figure 15.**
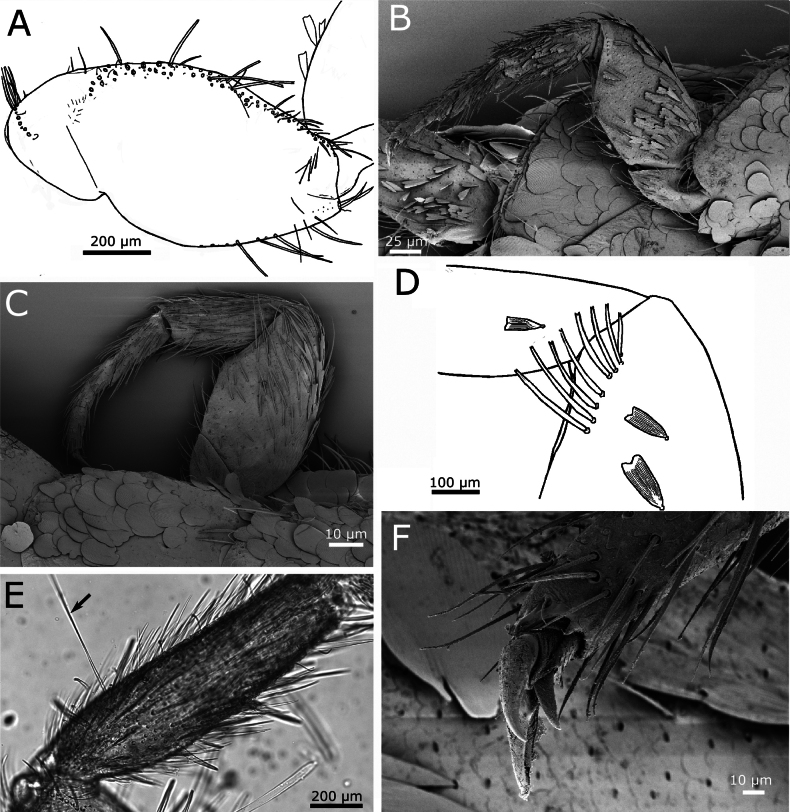
*Cactisma camanchaca* sp. nov., legs. **A.** Coxa of the first leg; **B.** Inner side (visible ventrally) of the first leg, showing subtriangular scales on femur and tibia; **C.** Outer side (visible dorsally) of the second leg, bearing only setae, except for the coxa which has rounded scales, and the anterior (dorsal) margin of the femur, where some subtriangular scales are inserted; **D.** Apical part of femur of the second leg with the subapical row of strong macrochaetae; **E.** Micrograph of a metatibia (holotype) with a dorsal trichobothrium (arrow); **F.** Pretarsus with claws.

**Figure 16.**
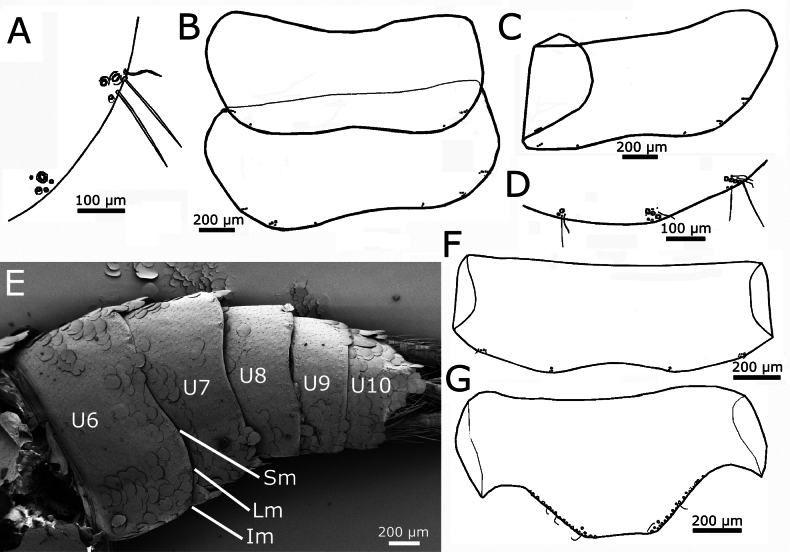
*Cactisma camanchaca* sp. nov., abdomen, dorsal. **A.** Chaetotaxy of urotergite I (right side), showing a small infralateral comb and a lateral group with insertion of only one strong macrochaeta; **B.** Urotergites I and II; **C.** Urotergite III; **D.** Chaetotaxy of the hind margin of urotergite III (right side, only insertions); **E.**SEM image of the posterior urotergites VI–X (marked as U6-U10); **F.** Urotergite VIII; **G.** Urotergite X. Abbreviation: Im: macrochaetae of infralateral combs, Lm: lateral macrochaeta, Sm: submedian macrochaeta.

**Figure 17.**
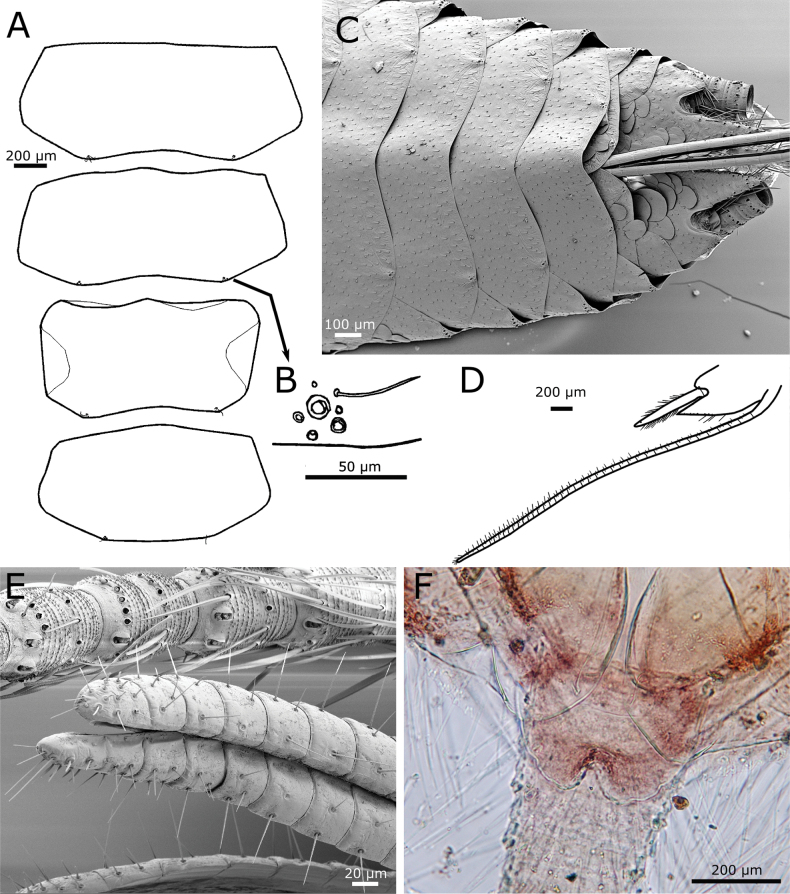
*Cactisma camanchaca* sp. nov., abdomen, ventral. **A.** Urosternites IV–VII; **B.** Insertion of a single macrochaeta and surrounding small setae on urosternite V; **C.** Posterior abdominal sternites including base of ovipositor; **D.** Lateral overview of ovipositor; **E.** Apical parts of gonapophyses VIII and part of a cercus in the upper half; **F.** Micrograph of epiproct and paraprocts observed behind the urotergite X. **A**, **B**, **D.** Schematic drawings; **C**, **E.**SEM images.

Figs 11, 12, 13, 14, 15, 16, 17

##### Type material.

***Holotype***: Chile • Antofagasta Region, Caleta el Cobre, Cuesta el Cobre, under dried cacti of the species Copiapoa (Pilocopiapoa) solaris (F. Ritter) F. Ritter; 24°17.83'S, 70°29.6'W; 750 m a.s.l.; 04.IX.2024. Leg. A. Zúñiga. 1♀* mounted on slide [MNNC]. ***Paratypes***: Same collection data as for holotype. One specimen mounted on slide: 1♀ juvenile [UCO]. Two specimens mounted on disc and gold coated: 1♀ [MZUC], 1♀ [MNNC] and two specimens preserved in ethanol 70%: 1♀ adult /1♀ juvenile [UCO].

##### Diagnosis.

As indicated for the new genus *Cactisma* gen. nov.

##### Description.

Habitus as in Fig. 11; body shape subcylindrical, more slender than *Lapidisma paposanum* sp. nov. Body length of holotype: 7.5 mm. Maximum body length observed in type series: 8.5 mm. Maximum preserved length antennae is 4.8 mm, but in live specimens antennae are as long as the body or slightly shorter. Epidermic pigment present, light brownish, only intense on margins of head, antennae, palps, urotergite X, styli, and caudal filaments. Macrochaetae smooth, bifid, hyaline, or pale yellowish. Body covered with oval or rounded scales (Fig. 12A); those with oval shape are the largest, reaching 180 × 110 µm, while the usual size of rounded dorsal and ventral scales is ~100 × 100 µm; the apical margin is rounded and the basal margin surrounds the socket forming more or less developed expansions (orbicular scales, Fig. 12B); all of them have very dense parallel ribs. Scales are also present on coxae, femora and tibiae and apparently absent from the remaining appendages; the shape of femoral and tibial scales is modified (see below).

Cephalic capsule with scales, except on clypeus and labrum. Frontal chaetotaxy extends from the periocular areas to the fronto-lateral corner; in the median part of the frons is an area with only a single row of macrochaetae close to the fronto-clypeal suture. The fronto-lateral fringe of macrochaetae has three or four series of macrochaetae, except for a lateral part anterior of the compound eyes where macrochaetae extend to the inner part of the frons forming a subtriangular tuft (Fig. 12C). The clypeus is mostly covered by a continuous broad fringe of macrochaetae consisting of six to eight irregularly arranged rows, including a row close to the frons; an area without setae on the margin close to the labrum. Labrum with a continuous fringe of densely packed setae of different length, some of which are bifid (Fig. 12D). Compound eyes usually with 12 ommatidia, sometimes only with 10 or 11 (Fig. 12D, E). Scape longer than pedicel, both apparently lacking scales and bearing a subapical ring of setae on their dorsal side. Antennal flagellum with chaetic, trichoid, and basiconic sensilla, as well as trichobothria; some basiconic sensilla are grouped in pairs (dyads) or grouped with trichoid sensilla, forming dyads or triads (Fig. 12G).

Mandibles with a well-developed incisive area with several teeth and a reduced molar area; below this area is a tuft of ~12 or 13 short macrochaetae and a characteristic long tuft on the basal part of the outer side with ~70 macrochaetae (Fig. 12F). Galea without apical tubercle. Lacinia with six or seven lamellate processes and a row of five or six apically bifid setae (Fig. 13A). Maxillary palp with its apical article slightly shorter than the antepenultimate, ~4.6–6.5× longer than wide and ~0.95–1.15× longer than the penultimate (Fig. 13B). This palp apparently lacks scales and bears some strong setae that are not clearly bifid apically on the apical region of the three basal articles. The apical article usually has three (two in some specimens) styloconic sensilla with a slender cylindrical style that is as long or slightly shorter than the distal width of the article, and four or five apical cones (Fig. 13C, D). In addition to the styloconic sensilla, a single cylindrical basiconic sensillum type C and four or five basiconic sensilla type B (Fig. 13D, E). All basiconic and styloconic sensilla are located at the distal part of the apical article; in addition, few coeloconic sensilla distributed over the complete length of article.

Labium as in Fig. 13F, with mentum clearly wider than long, the labial palp with its apical article not widened, the latter ~1.15–1.35× longer than wide and only slightly wider (~10%) than the maximum width of the penultimate article (Fig. 13G, H); it bears five papillae arranged in two curved lines, which can be interpreted as a pattern 3+2, but they can be better described as arranged in an oval shape (Fig. 13G, H). On the outer side of the apical article four or five basiconic sensilla type B and a single basiconic sensillum type C (Fig. 13I). In addition to the region-specific basiconic sensilla, several coeloconic sensilla are scattered over the entire apical article.

Pronotal collar continuous, with obliquely arranged rows of macrochaetae, containing four or five macrochaetae per row in the medio-lateral parts, but only one or two macrochaetae per row in the middle and at the anterolateral corners (Fig. 14A). Lateral margins of thoracic nota with several macrochaetae, either singly or in groups of two; some of them clearly marginal, while others are inserted at a more submarginal position; the macrochaetae that are not strictly marginal can be considered as reduced combs of only one or two macrochaetae. Two trichobothria likely present on the lateral margins of all thoracic nota; the position of these trichobothria is sometimes not clearly visible when they are broken off. On the pronotum, trichobothria have been observed at 0.49 (anterior trichobothrium) and 0.72 (posterior trichobothrium) of the lateral margins (Fig. 14A); on the mesonotum, at 0.6 and 0.78–0.82, respectively (Fig. 14B), and on the metanotum at 0.77 and 0.86, respectively (Fig. 14C). Posterior margins of thoracic nota without setae, except for the most posterior comb of the lateral margin, which is inserted at the posterolateral corner; these posterolateral combs consist of two large macrochaetae and one or two smaller setae inserted posteriorly to the macrochaetae, close to the margin of the notum (Fig. 14D, also visible in Fig. 14A−C).

Presternum of prothorax short, with two transverse irregular rows of macrochaetae (Fig. 13B). Thoracic sternites with parabolic shape, as in Fig. 14E−G. Prosternum (Fig. 14E) ~1.27× longer than wide, with convex posterior margin, slightly rounded; 1+1 antedistal combs with three or four macrochaetae each. Apart from antedistal combs, there is a row of thin setae inserted on both lateral margins from the base to the level of these combs (setae are absent on anterior and posterior margins). Mesosternum (Fig. 14F) similar in shape to the prosternum, with 1+1 antedistal combs with four or five macrochaetae each, its lateral margins bearing setae only in the posterior 1/2, anterior to the position of combs. Metasternum (Fig. 14G) shorter, slightly wider than long (ratio length/width ~0.93), its posterior margin straight in the middle, truncated; with 1+1 small posterior combs of two or three macrochaetae; marginal setae only on the posterior 1/3 of the lateral margin, directly anterior to the combs.

Coxae with rounded orbicular scales similar to those covering the body (but slightly smaller) and with an anterior row of densely packed macrochaetae, those on the apical part of the article form short oblique combs of two or three macrochaetae; the posterior margin of coxae has a row of very few widely spaced macrochaetae (Fig. 15A). Femora with small scales, subtriangular, with the apex truncated or bifid (with a median indentation) that cover the inner side of the article and the anterior margin of the outer side (Fig. 15B, C). Tibiae with scales similar to those of the femora, visible only on the inner side of the article (oriented ventrally). The size of these modified scales is smaller, femoral ones ~65 × 28 µm and those of tibiae ~45 × 22 µm. Apical margin of femora with a conspicuous row of long macrochaetae on their inner side (Fig. 15D). Tibiae with one or two dorsal and 2–4 ventral strong macrochaetae that are shorter than the width of the article. Metatibiae with a long trichobothrium inserted on the basal 1/2 of their anterior side (Fig. 15E). Ratio length/width of tibiae: ~2.3–3.3 for protibiae, 3.3 for mesotibiae and ~4 for metatibiae; metatibiae are 1.5–1.55× longer than protibiae.

First tarsomere of metatarsus ~0.58× the length of metatibiae, 1.4× longer than the corresponding tarsomere 2, 2.8× longer than tarsomere 3 and 1.65× longer than tarsomere 4. For the protarsus, these proportions are 0.45, 1.75, 2.25, and 2.1 respectively. Pretarsal claws smooth, as in Fig. 15F. Medial empodial claw shorter than 1/2 the length of lateral claws and faintly striated.

Urotergite I with 1+1 combs of two macrochaetae inserted infralaterally and an isolated comb in lateral position that can be accompanied by a smaller macrochaeta inserted obliquely (Fig. 16A). Urotergites II−VII with 3+3 combs of macrochaetae; infralateral combs consisting of 3–5 macrochaetae, lateral combs with two or three macrochaetae and sublateral combs small, with one or two macrochaetae (Fig. 16B−E); when they are formed by two, they are arranged obliquely, one of the two macrochaetae smaller and posterior to the other. Urotergite VIII with 2+2 combs of macrochaetae inserted on infralateral and submedian position, the infralateral with two or three macrochaetae and the submedian with the usual two macrochaetae arranged obliquely (Fig. 16F). Urotergite IX lacking setae. Urotergite X short trapezoidal, with setae inserted only on their lateral margins; the posterior margin straight (Fig. 16E, G).

Urosternite I without setae. Urosternites II−VII with 1+1 isolated macrochaetae in lateral positions (Fig. 17A), in the holotype one of the lateral macrochaetae is missing on urosternite VII. The isolated macrochaeta on each side is usually accompanied by several small setae (Fig. 17B). Coxites VIII with a comb of two setae on their posterior margin (Fig. 17C). Coxite IX only with marginal setae on their inner and outer processes; the single macrochaeta on the apex of the inner process cannot be considered as marginal. In females, inner process of the coxite IX ~1.6× longer than wide at the base and ~4–5× longer than the outer process (Fig. 17D). Only one pair of styli on the ninth segment; these styli are large, with dark pigment, and 2.2–2.3× longer than the inner process of the coxite IX. Ovipositor of primary type, very long (Fig. 17D), its apex as in Fig. 17E, surpassing the length of styli IX by 2.7–3.1× and the apex of coxites IX by ~7.5–8×. Gonapophyses with 43–45 divisions. Paraprocts and epiproct large, the apex of the epiproct reaching or even surpassing the level of the hind margin of the urotergite X, with a median indentation (Fig. 17F). Caudal filaments bearing bifid macrochaetae, trichobothria, chaetic and trichoid sensilla; apparently lacking scales (Fig. 17E). Male unknown.

##### Habitat and behaviour.

**Figure 18.**
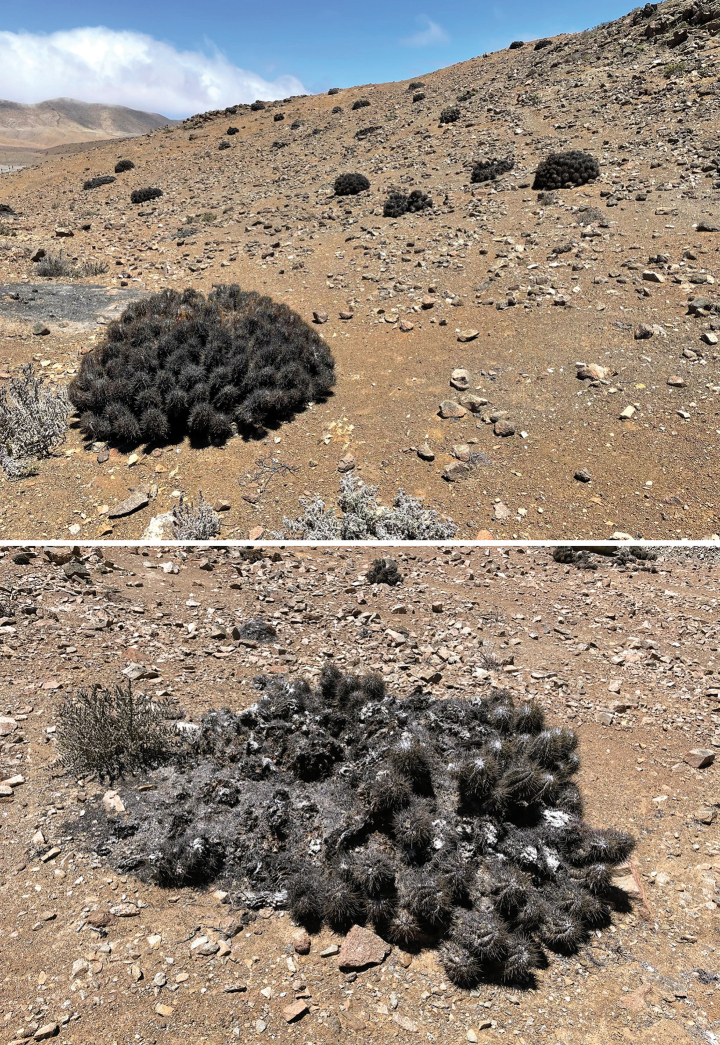
Habitat typical of *Cactisma camanchaca* sp. nov. at Cuesta el Cobre (Atacama Desert, Chile) with dried parts of Copiapoa (Pilocopiapoa) solaris (F. Ritter) F. Ritter.

Habitat similar to that described above for *L. paposanum*, but the type locality at higher altitude on the Coastal Cordillera, receiving more humidity from regular fog (camanchaca). The very scarce perennial vegetation is dominated by the cactus species Copiapoa (Pilocopiapoa) solaris (Fig. 18). Adults and juveniles were found together under dried and already rotting cacti (Fig. 18), and they escaped rapidly when disturbed.

##### Etymology.

The specific name *camanchaca* refers to the local name given to the fog that rises from the Pacific Ocean and regularly provides water to the coastal region of the Atacama Desert.

#### ﻿ Heterolepisma andinum

Taxon classificationAnimaliaZygentomaLepismatidae

(Silvestri, 1902)

2E11EB07-B5FD-5E56-AC74-579C5E722020

Figs 19, 20

Lepisma andina Silvestri, 1902: 230. Type locality: Argentina, Mendoza, Cacheuta.
Heterolepisma andina (Silvestri).—Escherich 1905: 64.
Isolepisma andina (Silvestri).—Wygodzinsky 1948: 219.

##### Examined material.

Argentina • La Rioja Province, Sanogasta, Cuesta de Miranda, under stones; 29°21.05'S, 67°46.94'W; 2000 m a.s.l.; 13. II. 2022. Leg. A. Zúñiga. 1♀* mounted on slide [MZUC].

##### Descriptive remarks.

The specimen used for morphological studies, collected in an area of Argentine Andes close to the border with Chile, matches the combination of characters attributed to *H. andinum* in the key of Stach (1933) and the few useful traits indicated in the obsolete original description provided by Silvestri (1902). It has one pair of styli, the urotergite X is short, the posterior margins of urosternites II−VI bear more than 1+1 macrochaetae (four or five macrochaetae), and the submedian combs of urotergites are composed of only one macrochaeta (a second smaller seta is inserted obliquely to the larger macrochaeta, which depending on the criterion, could be also considered as the presence of a comb of two macrochaetae different in size). This insect is tentatively treated here as *H. andinum*.

##### Other characteristics of this Argentine species are detailed below.

**Figure 19.**
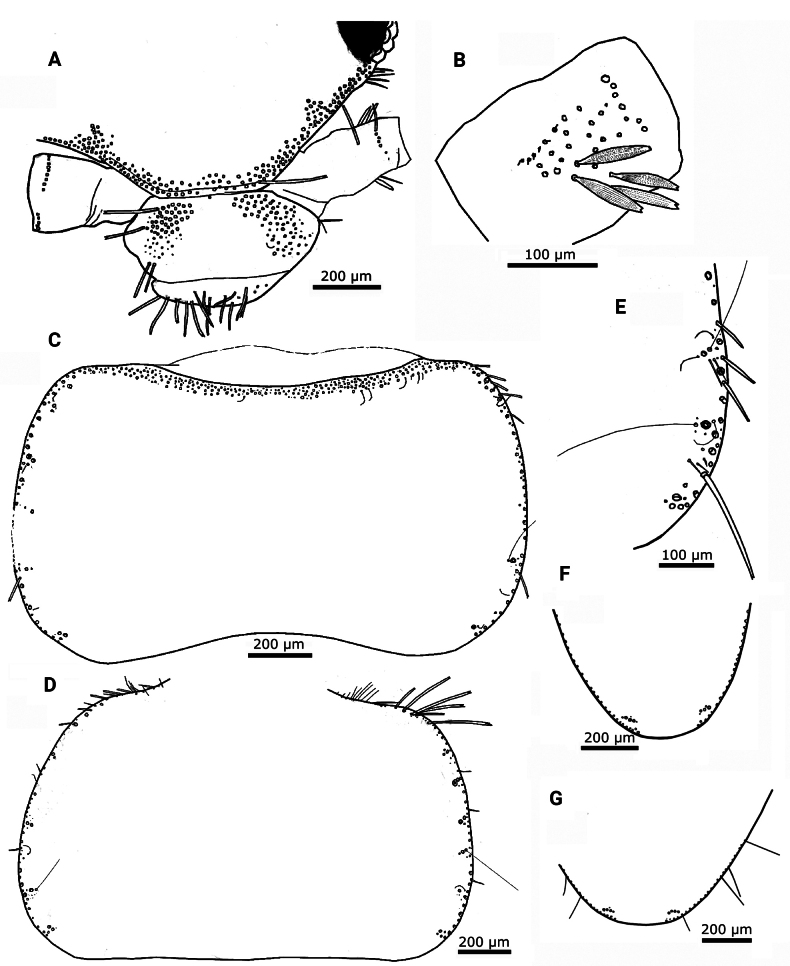
*Heterolepisma andinum*, schematic drawings of head and thorax. **A.** Chaetotaxy of frons, clypeus and labrum; **B.** Scales of scapus; **C.** Pronotum with preserved chaetotaxy on margins; **D.** Mesonotum with preserved chaetotaxy on margins; **E.** Postero-lateral right margin of metanotum, showing the position of anterior and posterior trichobothria; **F.** Hind margin of mesosternum; **G.** Hind margin of metasternum.

**Figure 20.**
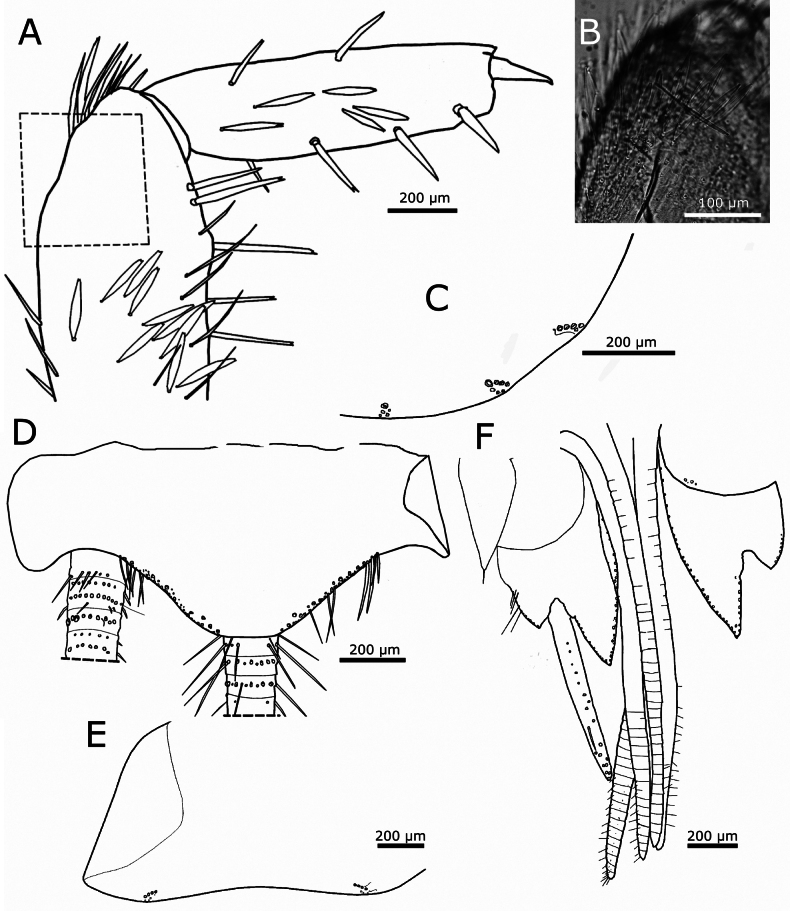
*Heterolepisma andinum*, schematic drawings of legs and abdomen. **A.** Metafemur and metatibia with position of macrochaetae and long setae (small setae are not represented), and few scales; **B.** Detail (micrograph) of the subapical area of the femur marked in **A.**, showing several femoral scales; **C.** Hind margin of the right lateral-infralateral part of urotergite III with insertions of macrochaetae arranged in combs (the one on submedian position reduced to one macrochaeta, surrounded by smaller setae close to the posterior margin); **D.** Urotergite X and base of caudal filaments (broken); **E.** Urosternite IV with insertions of macrochaetae arranged in 1+1 lateral combs; **F.** Genital area of the female specimen examined, showing coxites IX, one stylus inserted on one of these coxites, and its ovipositor. Divisions of gonapophyses are indicated only for areas where they are clearly visible. The posterior margins of coxites VIII are outlined to show that they lack insertions of styli.

Frontal fringe of macrochaetae continuous, without a clear median gap, approximately three or four rows of macrochaetae in the middle, with a wider triangular area laterally between frons and compound eyes (Fig. 19A). Clypeus with 1+1 lateral tufts of macrochaetae (Fig. 19A); scales not visible. Labrum with several bifid macrochaetae irregularly arranged in a transverse fringe. Scales on scapus lanceolate, with a small indentation at their apex (Fig. 19B); scales on pedicel not detected. Several types of sensilla have been observed on the flagellum (chaetic, trichoid and trichobothria). Apical article of the maxillary palp bearing three small styloconic sensilla with few short cones; scales not observed on maxillary palp. Pronotal collar continuous, with abundant macrochaetae arranged in two to five rows (Fig. 19C). Lateral margins of thoracic nota with several macrochaetae that in some cases are arranged in small combs of two or three macrochaetae and in some others they do not form clearly defined combs; in some positions isolated macrochaetae, which can be interpreted as reduced lateral combs; at the posterolateral corner there are 1+1 of such reduced combs of macrochaetae that consists of one to three macrochaetae and some small trichoid sensilla. Posterior margins of thoracic nota without setae (Fig. 19C, D). Two pairs of trichobothria on each notum; on the pronotum, only a single trichobothrium detected at 0.69 of the lateral margin; this is probably the posterior trichobothrium. On the mesonotum, the anterior trichobothrium at 0.59 of the lateral margins and the posterior trichobothrium at 0.75 (Fig. 19D). On the metanotum, anterior and posterior trichobothria are observed at 0.65 and 0.79 of the lateral margins, respectively (Fig. 19E). Thoracic sternites parabolic, with 1+1 antedistal combs of three or four macrochaetae each (Fig. 19F, G); these sternites are broken, and the prosternum is also bent on the slide, so their ratio length/width is not indicated. Scales of femora and tibiae lanceolate, with a small indentation at the apex (Fig. 20A, B); tibial scales slightly smaller than femoral ones. Urotergites I−IV with 3+3 combs, the infralateral comb with four or five macrochaetae, the lateral combs with three or four macrochaetae, and the submedian comb is reduced to one or two macrochaetae (Fig. 20C). Urotergites V−VII damaged, urotergite VIII with 2+2 combs (the submedian comb is missing). Urotergite IX apparently lacking setae; urotergite X short, convex, subtriangular to truncate, with the posterior margin almost straight (Fig. 20D), with some marginal macrochaetae that do not form visible combs. Urosternite I damaged; urosternites II−VI with 1+1 combs of three or four macrochaetae (Fig. 20E). Chaetotaxy of abdominal segment VII not clearly observed. Coxites VIII with a small comb of three macrochaetae, apparently lacking styli, so there is only one pair of styli on segment IX. Inner process of the coxite IX ~1.35× longer than wide and 3.6× longer than the outer process (Fig. 20F). The styli are ~2.3× longer than the inner process of the coxite IX. Ovipositor of the primary type, short, with ~32 or more divisions, surpassing the apex of styli only by 0.5× and surpassing the apex of the inner process of the coxite IX by 2.2× (Fig. 20F); the available specimen seems to be subadult (suggested by the short divisions of gonapophyses) so it is likely that the ovipositor is longer in adult females. Epiproct densely covered by macrochaetae. Caudal filaments broken, their basal divisions apparently without scales (Fig. 20D). Male specimens not available.

### ﻿Rearrangement of the subfamily Heterolepismatinae

According to the molecular data, the Australian *Heterolepisma* and *Vistrolepisma* (formerly *Visma*) seem to be sister to the South American Heterolepismatinae (see below), and this is congruent with the morphological set of characters analysed for the same clades. Therefore, the currently recognised genus *Heterolepisma* probably does not represent a natural group. The primary reference for defining the characteristics of *Heterolepisma* s. str. are Argentine species, since the type species of the genus, *H. pampeanum*, was described from Argentina. Since no recently collected specimens of *H. pampeanum* were available, we have used specimens that can be attributed to *H. andinum*, another species described from Argentina together with *H. pampeanum*. We can conclude that the Australian *Heterolepisma* species are different from the available *Heterolepisma* from Argentina (see relevant morphological differences comparing Tables 2, 3 with Tables 4, 5) and the name *Silvestrisma* gen. nov. is proposed for Australian species. The species *Silvestrisma michaelseni* (Silvestri, 1908), originally described as *Heterolepisma michaelseni*, is proposed as the type species of the new genus *Silvestrisma*, since it is the first described species of this group (Silvestri 1908). The diagnostic characters defining *Silvestrisma* gen. nov. are the following: frontal chaetotaxy with a median gap without setae or with 1+1 median macrochaetae; alternatively with a continuous fringe connecting lateral areas of macrochaetae. Lanceolate scales often present at least on femora, frequently on tibiae and scape (except for some species), appendages without triangular scales and only with rounded scales on coxae. Styloconic sensilla of the last article of maxillary palp usually flower-like (i.e., short, wide and with several branches), although it is slender in females of some species where the styloconic sensilla show sexual dimorphism. Papillae of the last article of the labial palp compact, 3+2 usually arranged in a curved cluster (two curved rows). Pronotal collar complete. Posterior margin of thoracic nota with 1–2 +1–2 macrochaetae. Lateral combs of thoracic nota with 0–3 macrochaetae. Thoracic sternites usually triangular or cordiform, with convex hind margin (sometimes parabolic and/or hind margin of metasternum slightly truncate). Urotergite I with 2+2 or 3+3 combs of macrochaetae (when there are 2+2, those on submedian position are missing). Urotergites II–VII with 3+3 combs of macrochaetae; submedian combs small, with one or two macrochaetae. Urotergite VIII with 2+2 or 3+3 combs of macrochaetae (when there are 2+2, those on lateral position are missing). Urotergite IX without setae or with few infralateral small setae (acute setae or reduced macrochaetae). Urotergite X long, parabolic to trapezoidal depending on whether its hind margin is convex or straight. Urosternite I glabrous or with one small median comb of macrochaetae. Urosternites II−VIII of males and II−VII of females with 1+1 isolated lateral macrochaetae or 1+1 small combs (usually with 2–5 macrochaetae packed together). One to two pairs of abdominal styli, sometimes one additional pair in females of some species. Parameres small, usually less than 1/2 the length of the inner process of coxite IX.

Differences between *Silvestrisma* gen. nov. and the remaining lineages of the subfamily Heterolepismatinae are presented in Tables 2–5 and commented upon in the Discussion. Table 1 includes the species that can be assigned to the genus *Silvestrisma* gen. nov. (all previously described from Australia), and those that remain as belonging to *Heterolepisma*. The species *Heterolepisma howense* Womersley, 1942 from Lord Howe Island can be assigned to this genus because their characters, detailed by Smith and Mitchell (2019), clearly match with the diagnosis above. The possible affinities of the remaining species are commented upon in the Discussion.

### ﻿Molecular analysis

The matrix of COI gene sequences considers 16 species of Lepismatidae (*L. paposanum* / *C. camanchaca* with sequences of 2/3 specimens, resp.) and *Maindronia* sp. as outgroup and has a length of 614 bp, of which 281 are polymorphic sites and 250 are parsimony informative. The substitution model selected by Bayesian Information Criteria was TIM2+F+G4.

**Figure 21.**
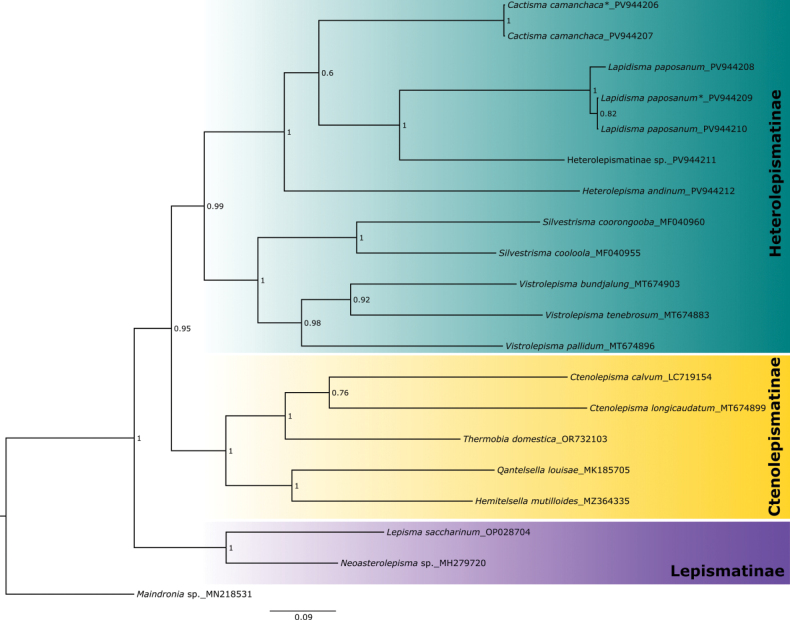
Bayesian phylogenetic tree based on COI gene sequences of Lepismatidae. Posterior probability values are given at the nodes. Sequences obtained from GenBank have the accession number added after the name. An asterisk (*) denotes the holotype specimen.

The tree obtained (Fig. 21) shows Lepismatinae (*Lepisma saccharina* Linnaeus, 1758 + *Neoasterolepisma* sp.) as sister to the remaining taxa (pp = 1), the latter with Ctenolepismatinae and Heterolepismatinae as sister groups (pp = 0.95). Ctenolepismatinae is divided (pp = 1) into a clade with *Thermobia domestica* (Packard, 1873) and (*Ctenolepisma longicaudatum* Escherich, 1905 + *Ctenolepisma calvum* (Ritter, 1910)) (pp = 1) and a clade including the Australian *Hemitelsella* and *Qantelsella* (pp = 1). Heterolepismatinae is divided into a clade with the Australian members as sister to the South American taxa (pp = 0.99). Within the Australian clade, *Silvestrisma* gen. nov. (i.e., *S. cooloola* (Smith, Mitchell, Lee & Espinasa, 2019) + *S. coorongooba* (Smith, Mitchell, Lee & Espinasa, 2019) is sister to the genus *Vistrolepisma* (pp = 1). Within the South American Heterolepismatinae, *H. andinum* is recovered as sister to the rest (pp = 1). The latter shows, with low node support (pp = 0.6), *C. camanchaca* as sister to an undescribed Heterolepismatinae from southern Chile and *L. paposanum* (pp = 1). Genetic distance between genera of South American Heterolepismatinae is ≥ 22%, while intraspecific distance of the material studied is ≤ 2% (see Suppl. material 2).

## ﻿Discussion

This study is a first step towards a better understanding of the diversity of the Heterolepismatinae of the world; our description of new genera also allows a more detailed comparison with the fairly well-studied fauna of Australia. The presented phylogeny is preliminary and based on a single gene only, but shows Heterolepismatinae as a monophyletic group and sister of Ctenolepismatinae. Lepismatinae appear as sister to Heterolepismatinae + Ctenolepismatinae and this does not support the hypothesis of Mendes (1991) who suggested Heterolepismatinae as the most plesiomorphic group and sister to the other subfamilies of Lepismatidae. Our morphological and molecular analyses also support the assignment of the two newly described Chilean genera to Heterolepismatinae. Although there are some morphological similarities between Australian *Vistrolepisma* and Chilean *Lapidisma* gen. nov., molecular data do not support a particular close relationship between Australian and Chilean Heterolepismatinae. The Australian taxa appear as sister to all analysed South American taxa, including *H. andinum*.

Heterolepismatinae now formally consists of six genera: *Heterolepisma*, *Vistrolepisma*, *Maritisma*, *Silvestrisma* gen. nov., *Lapidisma* gen. nov., and *Cactisma* gen. nov., which were all considered in our revision, including a first molecular analysis using COI gene sequences (except for *Maritisma*). Although this represents the most comprehensive revision of this subfamily to date, the taxonomy of Heterolepismatinae is far from being clarified. Some open taxonomic questions are discussed below.

Our morphological and molecular results show that *Silvestrisma* gen. nov. deserves to be separated from the topotypic South American *Heterolepisma*. The characters presented here for *H. andinum* are likely shared with *H. pampeanum*, except for the urosternal chaetotaxy, although the correct state of this latter trait in *H. pampeanum* is doubtful. Wygodzinsky (1948) and Stach (1933) mentioned 1+1 macrochaetae on urosternites of *H. pampeanum*, but the original description by Silvestri (1902) indicates 1+1 small combs of several setae on the same position, which is also the hypothesis preferred by Smith (2013), but this needs to be confirmed. Nevertheless, for the moment we attribute to *Heterolepisma* s. str. the characters presented in this work for *H. andinum*. The available specimen of this species is not in a perfect condition, so a complete redescription of the species *H. andinum* cannot be given (for example, we cannot confirm the state of characters such as the arrangement of papillae on the last segment of labial palps, shape and ratio length/width of thoracic sternites, chaetotaxy of urotergites I, V, VI, VII, IX, urosternites I, VII, shape of dorsal scales, length of antennae and cerci), but an important number of other characteristics of this insect from Argentina has been provided, in order to compare the species with more recently described taxa of Heterolepismatinae, including the new Chilean genera described in this work.

The differences of *Silvestrisma* gen. nov. to *Maritisma* and *Vistrolepisma* were detailed in Smith and Mitchell (2019) and in Smith et al. (2021), respectively and summarised in Tables 2, 3. Tables 4, 5 also list the differences to *Lapidisma* gen. nov. and *Cactisma* gen. nov.; the absence of chaetotaxy in the posterior margins of thoracic nota of the Chilean genera is one of the most remarkable differences, as well as the absence of lanceolate apically acute scales in the Chilean genera (except for the terminal filaments of *Lapidisma* gen. nov.), that are replaced by rounded or subtriangular, apically truncate scales. The presence of chaetotaxy in posterior margins of thoracic nota in *Silvestrisma* gen. nov. is also a difference to the Argentine *Heterolepisma* (confirmed in *H. andinum*), the latter corresponding to the genus *Heterolepisma* s. str. Further differences to *Heterolepisma* s. str. will be confirmed when the description of the type species of the genus (*H. pampeanum*) will be updated, but the characters observed in *H. andinum* and those available for *H. pampeanum* suggest that the different type of styloconic sensilla on the maxillary palps and the different shape of urotergite X, which is shorter in *Heterolepisma* s. str., as well as the probably larger parameres (characters shared with the new Chilean genera) can be presented as key characters to distinguish both genera.

Nevertheless, the characters described as variable in the diagnosis of *Silvestrisma* gen. nov. indicate that the genus could split into two or more genera or subgenera. Our molecular analyses of COI gene sequences of two species of *Silvestrisma* gen. nov. revealed a distinct genetic distance (>17%) (see Suppl. material 2) that could be correlated with the variable morphological traits (e.g., presence or absence of lanceolate scales, of median gap without setae in frons, of median comb on urosternite I).

*Heterolepisma zelandicum* (Tillyard, 1924), described from New Zealand as *Notolepisma zelandica*, could be congeneric with *Silvestrisma* gen. nov., and in this case the name *Notolepisma* would have priority for designating the Australian genus, but this species requires revision since the original description and the comments later given by Wygodzinsky (1961) do not provide enough information about the morphological differences or similarities with species from Australia.

The name *Isolepisma* Escherich, 1905 is also discarded for the new Australian genus of *Heterolepisma*. This name was used as a synonym of *Heterolepisma* and F. Silvestri used it for describing some species from America and other areas. The genus *Isolepisma* was introduced for the species *I. trisetosum* Escherich, 1905, a species that can be considered species inquirenda since the characters defining the species are very confusing and it has been cited from very separated geographic regions (Brazil, Angola, Indonesia) (Mendes 2011).

Further species described from other geographic regions (Caribbean area, Africa, etc.) as belonging to *Heterolepisma* should be analysed in future research to check the possibility of being congeneric with *Heterolepisma* s. str. or with other genera of Heterolepismatinae, i.e., *Maritisma* in Smith and Mitchell (2019) and *Visma* (now *Vistrolepisma*) in Smith et al. (2021), or new genera should be erected for them. Considering what is already known about the previously described species found outside Australia, we can already state that *H. annectens* from Juan Fernández Islands agrees very well with *Lapidisma* gen. nov. due to the absence of posterolateral macrochaetae on thoracic nota, the size of parameres and the number of pairs of styli, and it is proposed here to assign it to the genus *Lapidisma*.

Moreover, we can hypothesise that:

- The undescribed Heterolepismatinae from Talcahuano, Chile (36°43'S, 73°07'W), included in our phylogenetic tree (Fig. 21), is at least genetically, rather closely related to *Lapidisma* gen. nov., but this taxon still needs to be described morphologically to decide whether it is congeneric with *Lapidisma paposanum* sp. nov. or deserves its own genus, given the large genetic distance of more than 20% to *L. paposanum* (see Suppl. material 2).
- Species of *Heterolepisma* from the Asia-Pacific region (*H. japonicum* (Uchida, 1968), *H. mumfordi* (Silvestri, 1935), *H. rouxi* (Silvestri, 1915), and *H. tonga* Mendes, 2012) could belong to *Silvestrisma* gen. nov. because the characters described for these species are congruent with the above diagnosis.
- Species described from northern South America and the Caribbean (*Heterolepisma horni* Stach, 1933, *Heterolepisma insulare* (Banks, 1901), and *Heterolepisma serranoi* Mendes, 2011) very likely belong to an as yet undescribed genus that is clearly different from *Lapidisma* gen. nov., *Cactisma* gen. nov. and *Heterolepisma* s. str., which may be endemic to the Southern Cone of America. Northern species bear posterolateral macrochaetae on thoracic nota, but due the lack of data for several characters, these species should be revised to clarify their generic status. The only species with a more detailed description, *H. serranoi*, is similar to *H. andinum* (*Heterolepisma* s. str.) by the presence of lanceolate scales, but differs in the type of sensilla of maxillary palps, the shape of the urotergite X, and the chaetotaxy of urosternite I.
- Species of Heterolepismatinae described from southern Africa and neighbouring areas of the Indian Ocean (*Heterolepisma bisetosum* (Carpenter, 1916), *Heterolepisma exactum* (Silvestri, 1918), *Heterolepisma mossambicense* Mendes, 1993, and *Heterolepisma primafrum* (Silvestri, 1949)) could eventually belong to the same genus as the species in northern South America or to *Silvestrisma* gen. nov., since some characters are shared with them (lanceolate scales in more recently described species, presence of posterolateral macrochaetae on thoracic nota). However, since several relevant characters are not described for these species (e.g., chaetotaxy and scales of clypeus, scape, pedicel, styli, caudal filaments), they could also belong to a different genus exclusive to this geographic area. A close relationship with Argentine *Heterolepisma* s. str. is less probable because the presence of posterolateral macrochaetae on thoracic nota.

Kaplin (2023) mentioned the genus *Allacrotelsa* as belonging to the subfamily Heterolepismatinae, without providing detailed evidence to support this assumption. According to the phylogeny of Mendes (1991) based on 57 morphological characters, the genus *Allacrotelsa* is placed in the subfamily Lepismatinae as sister to the other members of this subfamily based on the synapomorphy “absence of setal collar on the anterior margin of the pronotum”. Later, Smith (2016) listed a number of shared characters of *Allacrotelsa* with Acrotelsatinae (e.g., cephalic chaetotaxy and unspecialised sensilla on the antennae), while Mendes (2018) reported a number of ancestral characters for *Allacrotelsa* also visible in Eocene fossils. Other characters are shared with most Lepismatinae, such as the occurrence of median combs on urosternites, and the absence of scales on femora (this absence in most genera of Lepismatinae has been confirmed by us). Molero-Baltanás et al. (2024) in their revision of Lepismatidae also discussed the position of *Allacrotelsa* and confirmed that the position of this genus is still not clear, leaving it provisionally in Lepismatinae. Thus, current data do not support the placement of *Allacrotelsa* in Heterolepismatinae.

Our present study focused on silverfish collected in a coastal fog oasis between Paposo and Cuesta el Cobre, a narrow strip of 80 km where we could find the two new genera of Heterolepismatinae. Molecular data from specimens of other Chilean regions show a fairly high diversity of Heterolepismatinae distributed along the coast from Talcahuano in the south to Antofagasta (23°38'S) in the north, not entering the Intermediate Depression or the Andes Cordillera. A survey in southern Peru also did not reveal any Heterolepismatinae, while they are present along the Argentine Precordillera from Mendoza to La Rioja and the Pampa, from Buenos Aires to Santa Cruz (Silvestri 1902; pers. obs.).

## Supplementary Material

XML Treatment for Lapidisma

XML Treatment for Lapidisma paposanum

XML Treatment for Cactisma

XML Treatment for Cactisma camanchaca

XML Treatment for Heterolepisma andinum

## ﻿Additional information

### Use of AI

No use of AI was reported.

### Author ORCIDs

Rafael Molero-Baltanás https://orcid.org/0000-0002-1489-3968

Álvaro Zúñiga-Reinoso https://orcid.org/0000-0002-7949-081X

Miquel Gaju-Ricart https://orcid.org/0000-0001-9236-1556

Reinhard Predel https://orcid.org/0000-0002-0202-6672

### Data availability

All of the data that support the findings of this study are available in the main text or Supplementary Information.

## Supplementary materials

Supplementary material 1 Aligned COI gene sequences in FASTA format that were used for phylogenetic reconstruction Data type docx Explanation note GenBank accession numbers have been added. https://binary.pensoft.net/file/1471806This dataset is made available under the Open Database License (http://opendatacommons.org/licenses/odbl/1.0/). The Open Database License (ODbL) is a license agreement intended to allow users to freely share, modify, and use this Dataset while maintaining this same freedom for others, provided that the original source and author(s) are credited.Rafael Molero-Baltanás, Álvaro Zúñiga-Reinoso, Miquel Gaju-Ricart, Reinhard Predel

Supplementary material 2 Information on the genetic distance of the sequences of COI genes used in this study Data type docx Explanation note * = holotype specimen. Bold numbers indicate observed intraspecific variability of the COI gene sequences. https://binary.pensoft.net/file/1471807This dataset is made available under the Open Database License (http://opendatacommons.org/licenses/odbl/1.0/). The Open Database License (ODbL) is a license agreement intended to allow users to freely share, modify, and use this Dataset while maintaining this same freedom for others, provided that the original source and author(s) are credited.Rafael Molero-Baltanás, Álvaro Zúñiga-Reinoso, Miquel Gaju-Ricart, Reinhard Predel
