# Supplementary material for: ﻿Morphological and molecular revision of the subfamily Heterolepismatinae (Zygentoma, Lepismatidae), with descriptions of two new genera from the Atacama Desert, Chile
Source: Zookeys. 2025 Nov 20;1260:233–78. doi: 10.3897/zookeys.1260.151902 (PMC12661332; doi:10.3897/zookeys.1260.151902)
Supplement: Supplementary material 1 — Aligned COI gene sequences in FASTA format that were used for phylogenetic reconstruction [file zookeys-1260-233_article-151902__-s001.docx]

**Supplementary material 1. Aligned COI gene sequences in FASTA format that were used for phylogenetic reconstruction. GenBank accession numbers have been added.**

>Maindronia_sp_MN218531 GCATAACTGGAACTTCCCTAAGCCTGCTAATCCGAGCAGAATTAGGGCAGCCAGGTAGACTAATTGGAGATGACCAAATCTATAACGTAATTGTCACAGCACATGCATTCATTATAATCTTCTTCATAGTTATACCAATTATGATTGGTGGCTTTGGGAACTGATTAGTACCCCTGATATTAGGTGCACCAGACATAGCATTCCCACGACTAAACAACATAAGCTTTTGACTACTACCCCCCTCATTAACCCTTCTATTATCAGGAAGAATTGTAGAAAGTGGTGCAGGAACTGGGTGAACTGTATACCCTCCCCTGTCAGCAGGGATTGCGCACGCTGGTGCATCAGTAGATCTATCAATTTTTTCATTACACTTAGCAGGTGTATCATCCATTCTAGGAGCAGTAAACTTTATTACCACAATCATCAATATACGAACCCCAGGAATAACAATAGATCGTACCCCTCTATTTGTGTGGGCAGTGTTTATTACAGCCATTTTATTACTCCTTTCATTGCCTGTCCTGGCAGGAGCCATTACTATACTATTGACAGACCGTAACTTAAATACCTCATTCTTTGACCCCTCAGGGGGGGGAGACCCAATTCTATAC

>Lepisma_saccharinum_OP028704 GAATAGTTGGAACATCATTAAGAATCTTAATCCGTATAGAATTAGGCCAACCAGGAAGTTTAATTGGAGATGACCAAATCTACAATGTAATTGTAACTGCACACGCATTTATTATAATTTTTTTCATAGTAATACCCATTATAATTGGAGGATTTGGAAACTGATTAGTGCCTTTAATAATTGGGGCCCCAGACATAGCCTTCCCACGATTAAATAACATAAGATTTTGATTACTACCCCCATCACTAACTCTACTATTATCAGGAAGAATTGTAGAAAGTGGTGCAGGAACTGGTTGAACAGTATATCCTCCTCTATCTGCAGGAATTGCACATGCTGGAGCCTCTGTTGACTTATCAATTTTTTCATTACATTTAGCAGGAGTATCCTCTATTCTAGGGGCAGTAAACTTTATTACGACAGTAATTAATATACGAACTATAGGAATATCTATAGAACGAGTACCATTATTTGTATGATCAGTAGTAATTACTGCAGTTCTACTGTTATTATCACTACCAGTCCTAGCAGGAGCAATTACCATATTATTAACAGATCGAAACTTAAATACCTCATTTTTTGATCCATCTGGTGGTGGAGATCCAATCTTATAT

>Neoasterolepisma_sp_MH279720 GTATAGTAGGAACCTCTCTAAGTATTTTAATTCGAATAGAATTAGGTCAGCCAGGAAGACTAATTGGAGACGACCAAATTTACAATGTAATTGTAACAGCACACGCATTTATTATAATTTTCTTCATAGTTATACCTGTTATAATTGGGGGGTTTGGTAATTGATTAGTTCCTCTAATATTAGGGGCCCCAGATATAGCATTCCCACGACTAAATAACATAAGATTTTGATTACTTCCACCATCACTCACTTTACTCTTATCAGGAAGAGTTGTAGAGAGTGGTGCAGGAACTGGATGAACAGTCTATCCTCCTCTTTCCGCAGGRATCGCCCATGCTGGAGCATCTGTTGACTTATCTATTTTCTCATTACATTTAGCAGGAGTATCCTCTATTCTAGGAGCAGTAAACTTTATTACAACAGTTATTAATATACGA?CAATGGGTATATATATAGAACGAATCCCCCTATTCGTTTGATCAGTAGTCATTACTGCAATCCTATTACTACTTTCTCTTCCAGTATTAGCAGGAGCAATTACTATATTACTAACAGACCGAAATCTCAATACCTCATTTTTTGACCCATCCGGGGGGGGAGATCCAATTCTATAC

>Ctenolepisma_calvum_LC719154 GAATAGTAGGAACCTCCCTAAGCCTACTCATCCGAGCAGAACTTGGCCGACCTGGAAGCCTTATCGGGGATGATCAAATTTATAATGTCATTGTAACAGCACACGCATTCATCATAATTTTTTTCATAGTTATACCAATTATAATTGGGGGATTTGGAAACTGACTAGTCCCCCTCATGCTTGGAGCCCCTGATATAGCATTTCCTCGTCTAAACAACATAAGATTCTGATTACTCCCCCCCTCTCTCACACTATTATTATCAGGAAGAATTGTAGAAAGTGGGGCAGGAACAGGATGAACCGTATACCCCCCCCTCTCTAGAGCAACTGCCCACGCAGGGGGCTCAGTGGACCTTTCCATCTTCTCCCTCCACCTTGCTGGAGTTTCCTCAATCCTTGGTGCCGTCAACTTTATTACAACGATTATTAACATACGAGCTCCAGGAATACTTATAGAACGAACCTCACTATTTGTATGATCTGTTTTCATCACCGCAATTCTTCTTCTCCTATCTCTTCCGGTTCTCGCCGGAGCAATTACTATACTCTTAACAGACCGAAACCTAAATACATCCTTCTTTGACCCTTCAGGGGGAGGAGACCCAATTCTTTAT

>Ctenolepisma_longicaudatum_MT674899 GGATAGTAGGAACCTCCCTAAGACTTCTCATCCGGGCCGAGCTAGGCCGACCAGGAAGTCTGATTGGAGACGACCAAATTTATAACGTAATTGTTACTGCGCATGCCTTTATCATAATCTTTTTCATAGTAATACCGATCATAATCGGGGGCTTTGGTAATTGGCTCGTCCCCCTAATATTAGGAGCTCCAGATATAGCCTTCCCCCGACTTAATAATATGAGATTTTGACTCCTTCCCCCGTCCCTAACTCTCCTATTAACTGGAAGAATTGTAGAGAGTGGTGCAGGAACAGGATGAACTGTTTATCCCCCCCTTTCCAACGCAATTGCCCACGCCGGGAGATCAGTCGACCTATCCATCTTTTCMTTACATTTAGCTGGTGTATCCTCGATCCTAGGCGCCGTAAATTTCATTACAACAGTAATTAATATACGAATACCAGGAATAACAATAGAACGTGTACCTCTATTTGTTTGGTCTGTTCTAATTACAGCCATTTTATTATTATTGTCCCTTCCAGTTCTAGCAGGAGCCATTACAATACTCCTAACAGACCGTAATCTAAACACCTCTTTTTTTGACCCAACAGGGGGTGGAGACCCCATCCTATAT

>Qantelsella_louisae_MK185705 GAATAGTAGGTACTTCTCTTAGTTTATTAATTCGAGCCGAATTAGGACGACCAGGAAGCCTAATTGGAGATGATCAAATCTATAATGTAATTGTAACAGCACACGCTTTCATTATAATCTTCTTTATAGTTATGCCCATTATAATTGGTGGCTTCGGAAATTGATTAGTACCTCTAATACTAGGGGCCCCAGATATAGCATTTCCCCGATTAAATAATATAAGATTTTGACTACTACCCCCATCATTAACCTTGCTTCTGACAGGAAGAATTGTAGAAAGTGGTGCCGGAACGGGGTGAACAGTTTATCCCCCCTTATCAGCAGCTATTGCTCACGCGGGGGCATCCGTTGACTTATCAATTTTCTCTCTCCACTTAGCCGGTGTTTCATCAATTCTTGGAGCTGTAAACTTCATTACTACAGTAATTAATATACGTTCACCAGGAATGACCATAGAACGAGTCCCTCTATTCACCTGATCTGTAATCATTACAGCAGTATTGCTCTTACTTTCTCTTCCTGTTCTAGCAGGGGCTATCACAATACTATTAACAGACCGAAATCTAAATACATCTTTCTTTGACCCAGCCGGGGGAGGAGATCCGATTCTCTAC

>Hemitelsella_mutilloides_MZ364335 GAATAGTTGGTACATCGCTAAGTCTCTTAATTCGAGCCGAATTAGGACGACCGGGAAGACTAATTGGCGACGATCAAATTTATAATGTAATTGTAACAGCTCACGCATTTATTATGATTTTCTTCATAGTTATGCCCATTATAATTGGGGGATTTGGAAATTGATTGGTYCCTCTAATGTTAGGTGCCCCAGATATAGCTTTCCCTCGATTAAATAACATAAGCTTTTGGCTTCTCCCTCCCTCACTTACCCTCCTATTAACAGGAAGAATTGTAGAAAGAGGTGCAGGTACAGGATGAACCGTTTATCCCCCACTCTCAGCAGCCATCGCTCATGCTGGAGCATCAGTAGACTTATCCATCTTTTCCCTTCATCTTGCTGGGGTATCATCAATCCTAGGTGCCGTAAATTTTATCACAACAGTAATTAACATACGATCACCAGGTATAACTATAGAACGAGTTCCTCTATTTACCTGATCAGTAGTTATCACAGCAGTACTATTACTGCTCTCCCTTCCCGTATTAGCAGGAGCCATTACAATATTATTAACAGATCGAAATTTAAATACATCCTTCTTCGACCCTGCAGGAGGGGGAGATCCTATCCTATAT

>Thermobia_domestica_OR732103 GAATAGTAGGAACATCACTAAGTCTTCTCATTCGAGCAGAACTTGGTCGACCAGGTAGTTTAATTGGTGATGATCAAATTTATAATGTAATTGTTACTGCTCATGCTTTCATTATAATTTTCTTCATAGTTATGCCAATTATAATTGGGGGCTTTGGTAATTGATTAGTACCTTTAATACTAGGAGCACCTGATATAGCATTCCCCCGTCTAAACAACATAAGATTCTGATTACTACCACCCTCACTAACCCTACTATTAACAGGAAGAATTGTAGAGAGTGGTGCGGGAACCGGATGAACTGTATATCCCCCTCTTTCCAGAGCCATTGCTCACGCCGGAGCCTCTGTCGACCTTTCTATTTTCTCCCTCCATCTAGCTGGTGTATCCTCAATTCTTGGGGCAGTAAATTTTATTACAACTGTAATTAACATACGAACTCCTGGAATAACTTTAGAACGAATACCATTATTTGTTTGATCTGTAGTAATTACTGCTGTATTACTTCTTTTATCTCTTCCCGTATTAGCAGGAGCTATTACAATACTCTTAACTGACCGAAATCTTAATACCTCCTTCTTTGATCCTTCAGGAGGAGGAGATCCAATTTTATAT

>Cactisma_camanchaca_Holotype_ PV944206 GCATAATTGGAACATCCCTGAGATTATTAATTCGAGCAGAACTTGGTCAACCAGGAAGACTAATTGGTGACGATCAAATTTATAACGTAATTGTAACAGCCCACGCATTTATTATAATCTTCTTTATAGTCATACCAATTATAATTGGAGGATTTGGTAATTGATTAGTCCCATTAATGCTAGGAGCACCAGATATGGCATTCCCTCGACTAAACAACATAAGATTTTGATTGCTCCCACCATCTCTAACCCTACTACTAACAGGCAGCATTGTAGAAAGCGGGGCGGGTACCGGATGAACAGTGTACCCGCCCCTATCAGCAAGAATCGCCCACGGGGGGGCATCCGTGGACCTTTCAATTTTTTCTCTTCACTTAGCAGGTGTATCTTCAATCCTCGGAGCAGTAAACTTTATTACAACAGTAATTAACATGCGGGCTGTAGGCATAACCTTAGAGCGAATACCCCTATTTGTTTGATCTGTAATTATTACAGCTGTATTATTATTATTATCTCTGCCAGTACTGGCTGGGGCCATCACAATACTACTAACTGACCGCAACCTAAATACATCATTCTTTGATCCGGTTGGGGGGGGTGATCCTATCCTCTAC

>Cactisma_camanchaca_PV944207 GCATAATTGGAACATCCCTGAGATTATTAATTCGAGCAGAACTTGGTCAACCAGGAAGACTAATTGGTGACGATCAAATTTATAACGTAATTGTAACAGCCCACGCATTTATTATAATCTTCTTTATAGTCATACCAATTATAATTGGAGGATTTGGTAATTGATTAGTCCCATTAATGCTAGGAGCACCAGATATGGCATTCCCTCGACTAAACAACATAAGATTTTGATTGCTCCCACCATCTCTAACCCTACTACTAACAGGCAGCATTGTAGAAAGCGGGGCGGGTACCGGATGAACAGTGTACCCGCCCCTATCAGCAAGAATCGCCCACGGGGGGGCATCCGTGGACCTTTCAATTTTTTCTCTTCACTTAGCAGGTGTATCTTCAATCCTCGGAGCAGTAAACTTTATTACAACAGTAATTAACATGCGGGCTGTAGGCATAACCTTAGAGCGAATACCCCTATTTGTTTGATCTGTAATTATTACAGCTGTATTATTATTATTATCTCTGCCAGTACTGGCTGGGGCCATCACAATACTACTAACTGACCGCAACCTAAATACATCATTCTTTGATCCGGTTGGGGGGGGTGATCCTATCCTCTAC

>Lapidisma_paposanum_PV944208 GCATAGTAGGAACCTCACTAAGTTTACTAATTCGGGCCGAACTAGGACAGCCCGGAAGACTAATCGGAGATGATCAAATTTATAATGTAGTAGTAACAGCCCATGCATTCATTATAATTTTCTTCATGGTCATACCGATCATAATCGGAGGGTTTGGTAACTGATTAGTCCCACTAATACTTGGCGCCCCAGACATAGCATTCCCCCGACTAAACAACATGAGATTCTGACTACTCCCCCCCTCCCTAACTCTACTACTAATAGGAAGAATTGTAGAAAGTGGTGCTGGCACCGGCTGAACGGTTTATCCTCCTCTTTCCGCCAGATTAGCTCACGGGGGCGCCTCAGTAGACCTCTCTATCTTCTCTCTCCACCTAGCGGGAGTATCGTCAATCTTAGGAGCAGTAAACTTTATCACTACAGTGATTAATATGCGGGCAGTTGGAATAACCTTAGAGCGCATACCATTATTCGTCTGATCAGTCATAATCACAGCTGTATTATTACTATTATCTCTGCCAGTACTAGCCGGCGCAATTACAATACTACTAACAGACCGAAACCTAAACACCTCATTCTTCGACCCCAGAGGGGGCGGGGACCCAATCTTATAT

>Lapidisma_paposanum_Holotype_PV944209 GCATAGTAGGAACCTCACTAAGTTTATTAATTCGAGCCGAACTAGGACAGCCCGGAAGACTAATCGGAGATGATCAAATTTATAATGTAGTAGTAACAGCCCATGCATTCATTATAATTTTCTTCATGGTCATACCGATCATAATCGGAGGGTTTGGTAACTGATTAGTTCCACTAATGCTTGGCGCCCCAGACATAGCATTCCCCCGACTAAACAACATGAGATTCTGACTACTCCCCCCCTCCCTAACTCTACTACTAATAGGAAGAATTGTAGAAAGCGGTGCTGGCACCGGCTGAACGGTTTATCCCCCTCTTTCCGCCAGATTAGCTCATGGGGGCGCCTCAGTAGACCTCTCCATCTTCTCTCTCCACCTAGCGGGAGTATCGTCAATCTTAGGAGCAGTAAACTTTATCACTACAGTGATTAATATACGGGCAGTTGGAATAACCTTAGAGCGCATACCATTATTCGTTTGATCAGTCATAATCACAGCTGTATTATTACTATTATCTCTGCCAGTACTAGCCGGTGCAATTACAATACTACTAACAGACCGAAACCTAAACACCTCATTCTTCGACCCCAGAGGGGGTGGGGACCCAATCTTATAT

>Lapidisma_paposanum_PV944210 GCATAGTAGGAACCTCACTAAGTTTATTAATTCGAGCCGAACTAGGACAGCCCGGAAGACTAATCGGAGATGATCAAATTTATAATGTAGTAGTAACAGCCCATGCATTCATTATAATTTTCTTCATGGTCATACCGATCATAATCGGAGGGTTTGGTAACTGATTAGTTCCACTAATGCTTGGCGCCCCAGACATAGCATTCCCCCGACTAAACAACATGAGATTCTGACTACTCCCCCCCTCCCTAACTCTACTACTAATAGGAAGAATTGTAGAAAGCGGTGCTGGCACCGGCTGAACGGTTTATCCCCCTCTTTCCGCCAGATTAGCTCATGGGGGCGCCTCAGTAGACCTCTCCATCTTCTCTCTCCACCTAGCGGGAGTATCGTCAATCTTAGGAGCAGTAAACTTTATCACTACAGTGATTAATATACGGGCAGTTGGAATAACCTTAGAGCGCATACCATTATTCGTTTGATCAGTCATAATCACAGCTGTATTATTACTATTATCTCTGCCAGTACTAGCCGGTGCAATTACAATACTACTAACAGACCGAAACCTAAACACCTCATTCTTCGACCCCAGAGGGGGTGGGGACCCAATCTTATAT

>Heterolepismatinae_sp_PV944211 GCATAGTAGGAACATCTCTTAGACTACTAATCCGAGCTGAACTTGGACAGCCCGGCAGATTAATTGGAGATGACCAAATCTATAATGTAGTAGTAACAGCCCACGCATTTATTATAATTTTCTTTATAGTTATGCCAATCATAATCGGAGGATTTGGCAACTGATTAGTCCCACTAATACTTGGTGCTCCCGATATAGCATTCCCTCGTCTTAATAACATAAGATTCTGACTATTGCCCCCTTCTTTAACCCTTCTTCTAATAGGAAGAATTGTAGAAAGTGGGGCCGGTACTGGATGAACAGTATACCCCCCACTTTCAGCGGGAATCGCTCACGGGGGGGCATCCGTAGACTTATCCATCTTCTCTCTCCATTTAGCAGGTGTCTCATCAATCCTTGGGGCTGTGAACTTTATTACCACTGTAATTAACATACGTGCAGTCGGCATAACATTAGAACGAATACCTCTATTTGTTTGATCAGTCGTTATTACAGCTGTATTATTACTCCTATCACTACCAGTTCTCGCTGGTGCTATTACAATATTATTAACAGACCGAAACCTAAATACATCATTTTTTGATCCAAGAGGGGGGGGTGACCCAATTTTATAT

>Heterolepisma_andinum_PV944212 GAATGGTGGGTACCTCCCTTAGACTATTAATTCGTGCTGAATTAGGGCAACCTGGCAGACTCATTGGAGATGATCAGATTTACAATGTAATTGTAACCGCCCACGCATTCATTATGATTTTCTTCATAGTTATACCAATTATAATTGGTGGCTTTGGTAATTGATTGGTCCCCCTAATGTTAGGCGCCCCGGATATAGCTTTCCCCCGATTAAACAACATAAGATTTTGATTATTACCCCCCTCCCTCACCCTCCTTCTAACTGGAAGATTTGTAGAAAGCGGGGCGGGGACTGGGTGAACAGTCTACCCACCACTCTCCGCCAGAATTGCTCATGGGGGAGCATCCGTTGACCTTTCAATCTTCTCCTTACACCTCGCCGGTGTCTCCTCTATTTTAGGTGCCGTAAACTTCATTACAACAGTAATTAACATACGAACAGTGGGTATGACATTAGAACGAATGCCCCTCTTTGTTTGATCAGTTATAATTACAGCCGTGCTTTTACTACTTTCACTTCCAGTACTAGCAGGAGCAATTACTATACTATTAACTGACCGAAATTTAAATACATCCTTCTTTGACCCGGCAGGGGGTGGAGACCCTATTCTGTAT

> Silvestrisma_coorongooba_MF040960 GAATAATTGGAACATCACTTAGACTACTAATCCGAGCCGAACTTGGTCAGGCAGGAAGACTAATTGGGAATGACCAAATCTATAACGTAATTGTAACCGCACAYGCCTTCATCATAATCTTCTTCATAGTAATACCAATTATAATTGGGGGATTTGGTAATTGACTAGTCCCCTTAATACTAGGRGCCCCAGAYATAGCATTCCCACGACTCAAYAACATAAGATTCTGACTTCTTCCCCCCTCCCTAACCTTAYTATTAACTGGAAGTATAGTAGAAAACGGAGCTGGAACAGGATGGACGGTTTACCCTCCCCTATCAGCAAACATCGCCCATAGAGGAGCTTCAGTAGACTTATCCATCTTCTCATTACATCTAGCAGGAGTTTCCTCAATTCTCGGGGCAGTAAACTTCATTACTACAATCATTAACATACGAATAGAGGGAATAACCTTAGAACGAGCTCCCCTTTTCGTATGATCAGTTATTATTACCGCAGTATTACTCCTACTATCTCTACCTGTACT----------------------------------------------------------------------------------------

> Silvestrisma_cooloola_MF040955 GCATAGTMGGAACCTCACTCAGTCTTCTTATCCGAACAGAACTAGGCCAACCAGGAAGACTAATCGGAAACGACCAAATCTACAATGTAATCGTAACAGCACATGCTTTCATTATAATCTTCTTTATAGTAATACCCATTATAATCGGAGGATTCGGCAATTGACTAGTACCCTTAATACTCGGAGCCCCAGATATAGCCTTCCCACGACTAAATAATATAAGATTCTGATTACTACCCCCCTCACTAACCTTATTATTAATAGGCAGCATAGTAGAAAATGGAGCCGGAACTGGTTGAACCGTATATCCACCCCTATCAGCAAATATCGCCCACAGCGGGGCCTCCGTAGACCTTTCAATCTTCTCCCTTCATCTAGCCGGARTCTCCTCAATCCTAGGGGCAGTAAACTTCATTACAACCATCATTAATATACGAACAGAAGGAATAACCCTAGAACGAACCCCCTTATTCGTCTGATCAGTGATCATTACYGCAATCCTCCTACTATTATCATTACCCGTCCTAGCAGGAGCAATCACAATATTACTCACTGACCGAAACTTAAATACATCTTTCTTCGATCCCTCAGGGGGAGGAGACCCAATCCTATAC

> Vistrolepisma_bundjalung_MT674903 GAATGGTAGGAACTGCACTAAGACTACTAATCCGAGCAGAACTCGGCCAACCAGGAAGACTAATTGGAAAYGACCAAATCTACAATGTAATCGTAACAGCTCACGCATTTATCATAATCTTCTTTATAGTAATACCAATTATAATCGGAGGATTCGGAAATTGACTAGTACCCCTAATATTGGGAGCCCCAGACATGGCATTTCCCCGATTAAACAACATAAGATTCTGATTATTACCCCCCTCACTAACACTATTATTAACTGGAAGAATAGTACAGAGTGGTGCAGGCACCGGATGAACTGTATACCCCCCACTCTCAGCGGGAATTGCGCACAGAGGAGCATCAGTAGATCTATCAATTTTTTCCCTACATCTTGCTGGGGTATCATCAATTCTAGGAGCACTGAACTTCATTACAACCGTAATCAACATACGAACCATAGGAATAACAATGGAACGAACCCCACTATTCGTCTGATCAGTAATTATTACTGCAGTGCTACTTCTACTATCCCTTCCAGTCCTCGCTGGAGCCATCACTATATTACTCACCGACCGCAACTTAAATACATCCTTCTTCAACCCCTGTGGGGGAGGAGACCCCATCTTATAC

> Vistrolepisma_pallidum_MT674896 GCATAGTAGGAACATCAATAAGACTCCTTATCCGAGCAGAACTTGGACAACCAGGAAGTCTAATTGGAAATGATCAAATTTACAATGTAATTGTAACAGCACACGCATTCTTAATAATTTTCTTTATAGTAATACCAATCATAATTGGGGGGTTCGGTAATTGATTAGTCCCATTAATACTAGGAGCACCAGATATGGCCTTCCCACGACTCAATAATATAAGCTTCTGATTACTACCACCATCCCTAACATTATTATTAACAGGGAGAATAGTAGAAAGTGGTGCAGGTACAGGATGAACCGTATATCCACCACTTTCATCTAGAATTGCGCACAGAGGAGCCTCAGTAGACCTTTCAATTTTTTCCCTACATTTAGCGGGTGTATCATCAATCCTAGGTGCCCTTAACTTCATTACAACCGTAATTAACATACGCACCAAAGGAATAACCCTAGAACGCACCCCATTATTCGTATGATCAGTAATTATTACAGCAGTATTACTCCTCTTATCCCTACCAGTACTAGCCGGAGCAATCACCATACTATTAACAGACCGAAACCTAAATACATCATTTTTTGACCCCTCTGGTGGTGGTGATCCAATCCTCTAT

> Vistrolepisma_tenebrosum_MT674883 GCATGGTAGGTACATCCCTAAGATTACTAATTCGCGCAGAATTAGGACAACCAGGAAGACTAATTGGTAATGACCAAATYTACAATGTAATTGTAACAGCTCACGCATTTATTATAATTTTCTTTATAGTTATACCAATTATAATTGGAGGATTTGGAAACTGATTAGTACCATTAATGCTAGGTGCACCAGATATAGCATTCCCTCGACTAAACAATATAAGATTCTGACTACTTCCCCCATCACTAACGCTATTATTAACAGGAAGAATAGTAGAAAATGGTGCAGGTACYGGATGAACTGTATACCCGCCGCTATCAGCTAGAATTGCACATAGTGGAGCATCTGTAGATCTTTCCATCTTCTCATTACATCTCGCCGGAGTTTCATCAATTTTAGGAGCACTAAATTTTATTACAACTGTAATTAATATACGAATAATAGGAATAACAATGGAACGAGTCCCTCTATTTGTATGATCTGTAATCATTACAGCAATTCTATTACTACTATCATTACCCGTCCTAGCAGGAGCCATCACCATATTATTAACAGATCGTAATTTAAACACCTCATTCTTTGAT------------------------------
