# Supplementary material for: ﻿Morphological and molecular revision of the subfamily Heterolepismatinae (Zygentoma, Lepismatidae), with descriptions of two new genera from the Atacama Desert, Chile
Source: Zookeys. 2025 Nov 20;1260:233–78. doi: 10.3897/zookeys.1260.151902 (PMC12661332; doi:10.3897/zookeys.1260.151902)
Supplement: Supplementary material 2 — Information on the genetic distance of the sequences of COI genes used in this study [file zookeys-1260-233_article-151902__-s002.docx]

**Supplementary material 2. Information on the genetic distance of the sequences of COI genes used in this study. * = holotype specimen. Bold numbers indicate observed intraspecific variability of the COI gene sequences.**

|  | **Species** | **GenBank** | **1** | **2** | **3** | **4** | **5** | **6** | **7** | **8** | **9** | **10** | **11** | **12** | **13** | **14** | **15** | **16** | **17** | **18** | **19** | **20** |
| --- | --- | --- | --- | --- | --- | --- | --- | --- | --- | --- | --- | --- | --- | --- | --- | --- | --- | --- | --- | --- | --- | --- |
| **1** | *Cactisma camanchaca ** | PV944206 | - |  |  |  |  |  |  |  |  |  |  |  |  |  |  |  |  |  |  |  |
| **2** | *Cactisma camanchaca* | PV944207 | **0.00** | - |  |  |  |  |  |  |  |  |  |  |  |  |  |  |  |  |  |  |
| **3** | *Lapidisma paposanum* | PV944208 | 0.19 | 0.19 | - |  |  |  |  |  |  |  |  |  |  |  |  |  |  |  |  |  |
| **4** | *Lapidisma paposanum** | PV944209 | 0.19 | 0.19 | **0.02** | - |  |  |  |  |  |  |  |  |  |  |  |  |  |  |  |  |
| **5** | *Lapidisma paposanum* | PV944210 | 0.19 | 0.19 | **0.02** | **0.00** | - |  |  |  |  |  |  |  |  |  |  |  |  |  |  |  |
| **6** | Heterolepismatinae sp. | PV944211 | 0.18 | 0.18 | 0.18 | 0.18 | 0.18 | - |  |  |  |  |  |  |  |  |  |  |  |  |  |  |
| **7** | *Heterolepisma andinum* | PV944212 | 0.21 | 0.21 | 0.22 | 0.21 | 0.21 | 0.21 | - |  |  |  |  |  |  |  |  |  |  |  |  |  |
| **8** | *Silvestrisma coorongooba* | MF040960 | 0.20 | 0.20 | 0.22 | 0.22 | 0.22 | 0.22 | 0.23 | - |  |  |  |  |  |  |  |  |  |  |  |  |
| **9** | *Silvestrisma cooloola* | MF040955 | 0.22 | 0.22 | 0.22 | 0.22 | 0.22 | 0.23 | 0.24 | 0.17 | - |  |  |  |  |  |  |  |  |  |  |  |
| **10** | *Vistrolepisma bundjalung* | MT674903 | 0.22 | 0.22 | 0.24 | 0.24 | 0.24 | 0.22 | 0.23 | 0.20 | 0.18 | - |  |  |  |  |  |  |  |  |  |  |
| **11** | *Vistrolepisma pallidum* | MT674896 | 0.19 | 0.19 | 0.21 | 0.21 | 0.21 | 0.21 | 0.24 | 0.20 | 0.18 | 0.18 | - |  |  |  |  |  |  |  |  |  |
| **12** | *Vistrolepisma tenebrosum* | MT674883 | 0.19 | 0.19 | 0.22 | 0.22 | 0.22 | 0.20 | 0.21 | 0.20 | 0.19 | 0.18 | 0.19 | - |  |  |  |  |  |  |  |  |
| **13** | *Ctenolepisma calvum* | LC719154 | 0.24 | 0.24 | 0.24 | 0.24 | 0.24 | 0.23 | 0.23 | 0.23 | 0.22 | 0.22 | 0.22 | 0.23 | - |  |  |  |  |  |  |  |
| **14** | *Ctenolepisma longicaudatum* | MT674899 | 0.24 | 0.24 | 0.23 | 0.23 | 0.23 | 0.24 | 0.24 | 0.23 | 0.24 | 0.24 | 0.23 | 0.23 | 0.22 | - |  |  |  |  |  |  |
| **15** | *Qantelsella louisae* | MK185705 | 0.20 | 0.20 | 0.23 | 0.23 | 0.23 | 0.21 | 0.21 | 0.22 | 0.21 | 0.23 | 0.22 | 0.21 | 0.23 | 0.23 | - |  |  |  |  |  |
| **16** | *Hemitelsella mutilloides* | MZ364335 | 0.22 | 0.22 | 0.23 | 0.23 | 0.23 | 0.22 | 0.20 | 0.23 | 0.24 | 0.22 | 0.23 | 0.19 | 0.22 | 0.22 | 0.19 | - |  |  |  |  |
| **17** | *Thermobia domestica* | OR732103 | 0.20 | 0.20 | 0.21 | 0.21 | 0.21 | 0.21 | 0.22 | 0.22 | 0.22 | 0.22 | 0.20 | 0.21 | 0.18 | 0.20 | 0.20 | 0.19 | - |  |  |  |
| **18** | *Lepisma saccharinum* | OP028704 | 0.22 | 0.22 | 0.23 | 0.22 | 0.22 | 0.22 | 0.22 | 0.21 | 0.20 | 0.21 | 0.19 | 0.17 | 0.23 | 0.22 | 0.21 | 0.20 | 0.19 | - |  |  |
| **19** | *Neoasterolepisma* sp. | MH279720 | 0.21 | 0.21 | 0.22 | 0.21 | 0.21 | 0.22 | 0.20 | 0.21 | 0.21 | 0.22 | 0.22 | 0.19 | 0.23 | 0.22 | 0.19 | 0.19 | 0.19 | 0.14 | - |  |
| **20** | *Maindronia* sp. | MN218531 | 0.22 | 0.22 | 0.23 | 0.24 | 0.24 | 0.22 | 0.22 | 0.22 | 0.22 | 0.20 | 0.23 | 0.19 | 0.22 | 0.21 | 0.21 | 0.22 | 0.22 | 0.20 | 0.19 | - |
